# Mechanistic Insights into the Formation of Complex Coacervates in Crowded Environments

**DOI:** 10.1021/acs.biomac.6c01306

**Published:** 2026-08-21

**Authors:** Shanta Biswas, Sai Venkatesh Pingali, Katie T. Tran, Qingqiu Huang, Xiaobing Zuo, Amy Y. Xu

**Affiliations:** † Department of Chemistry, 5779 Louisiana State University, Baton Rouge, Louisiana 70803, United States; ‡ Neutron Scattering Division, 6146 Oak Ridge National Laboratory, Oak Ridge, Tennessee 37830, United States; § Cornell High Energy Synchrotron Source (CHESS), 5922 Cornell University, Ithaca, New York 14853, United States; ∥ X-Ray Source Division, Advanced Photon Source, 1291 Argonne National Laboratory, Lemont, Illinois 60439, United States

## Abstract

Complex coacervates are widely used as model systems for studying biomolecular condensate formation, yet how intracellular crowding influences coacervation mechanisms remains unclear. Here, we investigate a model protein–polymer coacervate system in dilute and crowded environments to elucidate its formation mechanism. In dilute solutions, protein–polymer complexes gradually grow into larger assemblies as their net charge approaches neutrality. Under crowded conditions, excluded-volume effects force polymer chains into highly coiled conformations, creating a dense polymer network before the protein is introduced. Protein incorporation into this network relieves conformational tension and triggers the formation of protein–polymer networks that act as scaffolds for coacervates, bypassing the gradual charge-driven assembly seen under dilute conditions. Despite this mechanistic difference, crowders are found to be excluded from the dense phase, yielding comparable mesoscale structures under both conditions. Overall, this work suggests how condensates may adapt their assembly mechanisms within complex cellular environments.

## Graphical abstract

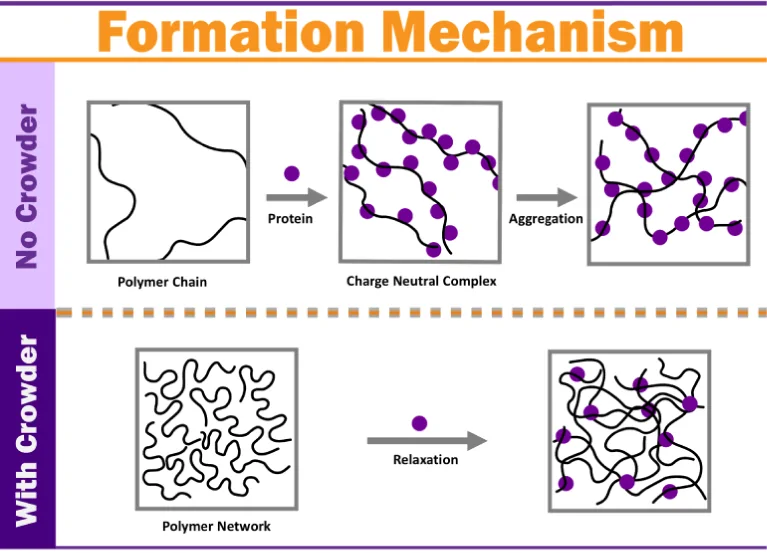

## Introduction

Membraneless organelles (MLOs) are biomolecular condensates found in living cells that are not enclosed by a lipid membrane. They appear as liquid-like droplets that are often concentrated with specific proteins and nucleic acids.^1–3^ These droplets are formed through liquid–liquid phase separation (LLPS) driven by electrostatic^4^ and other weak intermolecular interactions^5–7^ between these biomolecules.^4–9^ Despite their biological importance, the molecular mechanisms governing the formation of these cellular structures, particularly within the crowded cellular environment where they naturally occur, remain incompletely understood.^10–12^ To understand the physicochemical features of biomacromolecules that dictate their phase separation behavior, as well as the properties of the resulting liquid droplets, complex coacervates have been widely used as model systems.^13,14^ Like membraneless organelles, complex coacervates are formed via LLPS driven by electrostatic and other noncovalent interactions between charged macromolecules and appear as micrometer-sized liquid droplets that can selectively concentrate specific macromolecules.^15–17^ The validity of complex coacervates as model systems for studying membraneless organelles has been discussed in detail by Yewdall and coworkers.^13^

Complex coacervates can be formed by changing ionic strength, pH, temperature, macromolecular concentration, or by varying the stoichiometric ratio of the interacting macromolecules.^18–24^ The formation mechanism of coacervates depends on how such phase separation is induced, since different induction methods can lead to different assembly pathways. In studies where coacervation is induced by titrating one charged macromolecule into another (i.e., a change in stoichiometry), a well-defined sequential mechanism has been established. At first, soluble intra- and interpolymer complexes are formed through electrostatic interactions, as these complexes approach charge neutrality,^25^ electrostatic repulsion between them is eliminated, allowing them to coalesce extensively and ultimately form a dense coacervate phase in equilibrium with a dilute supernatant.^22,23,25–28^ While this mechanism has been well documented for many protein–polymer systems, it has been established for systems prepared under dilute conditions, where one macromolecular solution is titrated into another in the absence of crowding. Since MLOs are mostly formed within the highly crowded cellular environment, incorporating macromolecular crowding into complex coacervate model systems provides a more physiologically relevant framework for studying condensate formation. By examining coacervation under crowded conditions, it becomes possible to uncover assembly mechanisms that more closely reflect those operating inside living cells, thereby advancing our understanding of how biomolecular condensates form in their native cellular environment.

In this study, we used a model protein–polymer coacervate system consisting of bovine serum albumin (BSA) and polydiallyldimethylammonium chloride (PDADMAC) to examine the conformation and assembly of these macromolecules under both dilute and crowded conditions. This approach allows us to uncover distinct coacervate formation mechanisms under both dilute and crowded conditions. Polyethylene glycol (PEG) and Ficoll were selected as the macromolecular crowders. PEG is a linear polyether, while Ficoll is a highly branched copolymer of sucrose and epichlorohydrin, adopting a Gaussian coil and semirigid spherical conformation (Figure S1 in the Supporting Information), respectively. Both crowders are electrically neutral and were confirmed to have negligible interactions with BSA and PDADMAC by isothermal titration calorimetry (ITC) experiments (see Figure S2 in the Supporting Information). Our results show that under dilute conditions, coacervation proceeds through the gradual, charge-dependent assembly of soluble protein–polymer complexes, whereas under crowded conditions, excluded-volume effects imposed by the crowders drive PDADMAC chains into highly coiled conformations and force them into close proximity, which further leads to a dense polymeric environment that primes the system for coacervation upon protein addition. As soon as BSA molecules are introduced into the system, these preorganized polymer networks serve as scaffolds for near-instantaneous coacervate formation, bypassing the strict requirement for charge neutrality that governs coacervation under dilute conditions. Furthermore, although both PEG and Ficoll are excluded from the dense coacervate phase, their presence in the coexisting supernatant significantly promotes droplet coalescence, giving rise to larger droplets with broader size distributions. This demonstrates that the dense and dilute phases remain in active, dynamic communication after phase separation and that the composition of the supernatant can determine the droplet behavior in the dense phase. Overall, our findings suggest that macromolecular crowding reshapes the pathway of complex coacervation, lowers the macromolecular concentration threshold for phase separation, and offers new mechanistic insights into how biomolecular condensates may form more readily and adaptively in a crowded cellular environment.

## Experimental Section

### Materials

Bovine serum albumin (BSA) (Cat No. BP9706100) was purchased from Fisher Scientific, USA. Poly­(diallyldimethylammonium chloride) (PDADMAC) (400–500 kDa, Cat No. 409030), sodium chloride (Cat No. S9888), ficoll (70 kDa, Cat No. P2878), polyethylene glycol (PEG) (20 kDa, Cat No. P5413), and poly­(sodium 4-styrenesulfonate) (PSS) (200–300 kDa, Cat No. 561967) were purchased from Sigma-Aldrich, USA. Ultrapure water was used to prepare all of the solutions. All other reagents were of analytical grade and used without further purification.

### Preparation of BSA and PDADMAC Stock Solutions

In this study, BSA-PDADMAC complex coacervates were prepared under three different solution environments: 50 mM NaCl, 50 mM NaCl with 3 mM Ficoll (21% (w/v)), and 50 mM NaCl with 3 mM PEG (6% (w/v)). Here, Ficoll and PEG were employed as macromolecular crowding agents at concentrations sufficient to occupy a substantial fraction of the solution volume (Figure S1in the Supporting Information). These solution environments are referred to as NaCl, Ficoll, and PEG, respectively, throughout the manuscript for clarity. BSA stock solutions (∼100 mg/mL) were prepared by dissolving BSA powder in each solution and were allowed to solvate at 4 °C overnight to ensure complete hydration. PDADMAC stock solutions were prepared at 2 mg/mL (0.0044 μM) in each of the three solution environments.

### Turbidimetric Titration

In this experiment, 2 mL of PDADMAC solution at a concentration of 2 mg/mL was pipetted into a clean 1 cm path length cuvette, and a concentrated BSA solution was gradually added in 1–10 μL aliquots. At a critical protein concentration, the solution became visibly turbid, indicating the onset of coacervation. Turbidity was used to qualitatively measure the extent of coacervation as a function of the protein-to-polymer molar ratio. After each addition, the transmittance (*T*) of the mixture was measured at 450 nm at 20 °C using a UV–vis spectrophotometer (UV 1600 PC Spectrophotometer, VWR, USA) and subsequently converted to turbidity using standard mathematical relations-

1 Turbidity=100−%T

where

2 $$T=\frac{I_{t}}{I_{o}}$$

The calculated turbidity was plotted as a function of the increasing BSA-to-PDADMAC molar ratio (*r*). This analysis was performed under NaCl, Ficoll, and PEG solution conditions. In a separate set of experiments, the concentration of BSA was systematically increased to 50, 100, and 150 mg/mL and titrated against PDADMAC solutions at 2, 4, and 6 mg/mL, respectively. In all cases, control measurements confirmed that neither BSA nor PDADMAC, individually or in combination with Ficoll or PEG, exhibited measurable turbidity at 450 nm. Therefore, the observed turbidity changes were attributed directly to BSA-PDADMAC complex coacervate formation.

### Preparation of BSA-PDADMAC Complex Coacervates

The turbidimetric titration described above allowed us to determine the critical molar ratio at which BSA and PDADMAC begin to form complex coacervates. In order to prepare a coacervate at a fixed composition (*r* = 35, corresponding to a mass ratio of 5), calculated volumes of BSA and PDADMAC stock solutions were mixed. Upon mixing, the solution immediately turned turbid, indicating phase separation. The turbid mixtures were subsequently centrifuged at 4500 rpm for 20 min at 20 °C, yielding two optically distinct phases: a dense coacervate phase at the bottom and a dilute supernatant phase at the top. The resulting samples were stored in the same 15 mL tubes used for preparation, securely capped, sealed with parafilm, and maintained at 4 °C until further use.

### Protein Concentration Measurements

The concentration of BSA was determined by measuring the absorbance of the solution at 280 nm using a UV–vis spectrophotometer (UV-1600 PC Spectrophotometer, VWR, USA), with a molar extinction coefficient of 43824 M^–1^ cm^–1^. The volume and BSA concentration of the supernatant phase were measured after centrifugation, and the amount of BSA in the coacervate phase was determined by subtracting the BSA remaining in the supernatant from the total known amount added. Finally, the volume and mass of BSA within the coacervate phase were used to calculate the concentration of BSA in the coacervate phase.

### PDADMAC Concentration Measurements

The concentration of PDADMAC was determined by measuring the solution transmittance (%*T*) at 450 nm using a UV–vis spectrophotometer (UV-1600 PC Spectrophotometer, VWR, USA). A calibration curve was constructed by preparing PDADMAC standard solutions ranging from 0.05 to 0.4 mg/mL in each of the three solution conditions. To each 1 mL of the standard solution, 32 μL of negatively charged PSS was added, which interacted with positively charged PDADMAC, resulting in a turbid solution. The transmittance of the suspension was measured at 450 nm and converted into turbidity using eqs 1 and 2 The buffer solution without PDADMAC was used as the blank. The turbidity values were plotted against the concentration of PDADMAC to generate a calibration plot (Figure S3) for each buffer condition. In order to determine the concentration of PDADMAC in the supernatant, the collected supernatant was appropriately diluted in order to keep the transmittance within the calibration range after adding the same volume of PSS. The resulting turbidity was used to calculate the concentration of PDADMAC in the supernatant. Given the known volume of the coacervate phase and the total amount of PDADMAC initially added to prepare the coacervate, the concentration of PDADMAC in the coacervate phase was determined by subtracting the amount remaining in the supernatant from the total amount added.

### Small-Angle Neutron Scattering (SANS)

Small-angle neutron scattering experiments were performed on the Bio-SANS instrument^29^ in the High Flux Isotope Reactor (HFIR) at Oak Ridge National Laboratory (ORNL). The configuration consisted of a neutron wavelength of 6 Å and a detection system with three detector arrays. The sample-to-detector distance of the main detector array was set to 15.5 m, the midrange detector located at 4 m from the sample was rotated to 1.0°, and the wing detector located at 1.13 m from samples was rotated to 5.5° to achieve a *q* range of 0.003 A^–1^ to 0.85 A^–1^ in a single exposure. Samples were prepared in 100% D_2_O and 40% D_2_O and loaded into demountable 1 mm thick titanium cells for measurements. The SANS data reduction was performed using ORNL facility developed software “drtsans”^30^ to convert 2D images to 1D data of absolute-scale scattering intensity, *I*(*q*), as a function of the magnitude of the scattering vector *q* (eq 3). The scattering vector *q* is defined as:

3 $$q=\frac{4\pi }{\lambda }\sin\left(\theta \right)$$

where λ is the wavelength and 2θ is the scattering angle. For a system containing a monodisperse, homogeneous, and isotropic dispersion of spherical particles, *I*(*q*) can be expressed as-

4 $$I\left(q\right)=\phi {\left(\Delta \rho \right)}^{2}V_{P}P\left(q\right)S\left(q\right)$$

where ϕ is the volume fraction of the particles, *Δρ* is the difference in scattering length density between the scattering particles and the solvent, *V*_*p*_ is the volume of the particle, *P­(q)* is the form factor providing information on the size and shape of the scattering object, and *S­(q)* is the structure factor that is related to the spatial arrangements of particles and thus contains information on the interparticle interactions. Data analysis of PDADMAC in NaCl and coacervate samples in 40% D_2_O was performed using the NCNR analysis macro package built into IgorPro software.^31,32^ The effective concentration of PDADMAC was calculated in both Ficoll and PEG environments, and measured on the same beamline. The data were fitted using the hybrid model developed in SASView.^33^ Here, a 6 mg/mL (0.0132 μM) PDADMAC solution was measured in NaCl condition. The scattering profile in NaCl was analyzed by fitting the profile using the power-law model. The scattering intensity *I*(*q*) is calculated as:

5 $$I\left(q\right)=Aq^{-m}+background$$

Here, *A* is the scaling coefficient. Power law exponent *m* provides insights into the bulk or surface morphology of the scattering object,^34^ where *m* = 1 and 2 indicate conformations of a rigid rod and a randomly flexible Gaussian coil, respectively. *m* = 3 and 4 indicate rough and smooth surface morphologies, respectively; resembling the dense polymeric particles,^35^ in this study.

In order to better fit the scattering profile measured from a concentrated PDADMAC solution (prepared at a concentration representing the effective local concentration of PDADMAC within the Ficoll- and PEG-crowded environments), a hybrid model combining the correlation length model with a hard-sphere structure factor was used. In the crowded interior, the effective concentration of PDADMAC increases, leading PDADMAC to adopt an arrangement forming self-similar “blobs” that exhibit internal correlation. Here, each blob is considered as an imaginary hard sphere, and therefore the hard-sphere structure factor (*S­(q)*) was taken into account for repulsive interactions among these blobs, providing the average inter-blob distance. The correlation length model, described by a Lorentzian function, accounts for local density fluctuations of charged segments along the chain which provides an estimate of the characteristic blob size. The overall scattering intensity *I*(*q*) is then expressed as:

6 $$I\left(q\right)=I{\left(q\right)}_{correlation\;length}\cdot S\left(q\right)+background$$

The corresponding equation for correlation length model is:

7 $$I{\left(q\right)}_{Correlation\;length}=\frac{A}{q^{n}}+\frac{B}{1+{\left(\xi q\right)}^{m}}+background$$

The first term $\frac{A}{q^{n}}$ represents Porod scattering from polymer clusters (exponent = *n,*) and the second term, $\frac{B}{1+{\left(\xi q\right)}^{m}}$ is a Lorentzian function describing scattering from polymer chains (exponent = *m*), particularly indicating the conformation of the polymer. For SANS analysis, mean-field theory is typically applied, which describes the scattering arising from concentration fluctuations in a polymer solution at equilibrium. Under this framework, the Ornstein–Zernike (O–Z) equation, which describes concentration fluctuations at high *q*,^36^ can be replaced with the Lorentzian function in eq 7, which can be presented as:

8 $$I{\left(q\right)}_{Correlation\;length}=\frac{A}{q^{n}}+\frac{I_{oz}\left(0\right)}{1+{\left(\xi q\right)}^{m}}+background$$

Here, the exponent *n* defines the geometry of the scattering moiety present in a given system. For instance, *n* = 1, 2, and 4 correspond to the geometrical shapes of rod, Gaussian coil, and hard-sphere conformations, consisting of a smooth surface, respectively.^37^ The second term $\left(\frac{I_{oz}\left(0\right)}{1+{\left(\xi q\right)}^{m}}\right)$ is the Ornstein–Zernike (O–Z) function describing the scattering from semidilute polymer chains in a good solvent at equilibrium, which has led to a form of the structure factor of concentration fluctuations at high *q*.^36,38^ *I*_*OZ*_ *(0)* is the extrapolated structure factor at zero wavenumber, and ξ is the correlation length of the density fluctuations within the imaginary polymer blob^39^ in this study.

Besides these, coacervate scattering profiles measured in 40% D_2_O show two power-law slopes, with a crossover between regions with different slopes. The presence of these two regions with different slopes indicates a transition from individual polymer conformations to larger aggregated particles.^40^ The scattering intensity *I*(*q*) is calculated as:

9 $$I\left(q\right)=\{\begin{array}{l} Bq^{-m_{1}}+background,q\leq q_{c}\\ Cq^{-m_{2}}+background,q\geq q_{c} \end{array}$$

Here, *B* and *C* are the scaling coefficients in the low- and high-*q* regions, respectively. Power-law exponents *m*_1_ and *m*_2_ provide insights into the bulk or surface morphology of the scattering object^34^ at low- and high-*q*, respectively. Similar to the power-law model, *m* = 1 and 2 indicate the polymer conformation as a rigid rod and a randomly flexible Gaussian coil, respectively. Exponents of 3 and 4 indicate rough and smooth surface morphology, respectively, of the dense polymeric networks.,^35^ Here, *q*_*c*_ is the location of the crossover point from one slope to another. In this case, *m*_1_ represents local polymer conformation, while *m*_2_ represents the network structure of the aggregate polymeric particle. The fit parameters obtained from SANS measurements based on eqs 5–9 are found in Table S1 of the Supporting Information.

### Dynamic Light Scattering

Zeta potential (ζ-potential) measurements were used to correlate the charge status of the BSA-PDADMAC complexes with the evolution of turbidity. Here, the PDADMAC solution was initially measured, and the ζ-potential was recorded. BSA solution was then added incrementally to this PDADMAC solution, with the ζ-potential recorded after each addition to monitor the change in the charge status of the sample with turbidity. ζ-potential measurements were performed using a dynamic light scattering (DLS) instrument (Litesizer 500, Anton Paar, USA), in which samples were loaded into a cuvette followed by insertion of a Univette, allowing us to determine the particle electrophoretic mobility under an applied electric field. The electrophoretic mobility (μ) was calculated according to the following equation-

10 $$\mu =\frac{\nu }{E}$$

where ν is the drift velocity of a dispersed particle (m/s) and *E* is the applied electric field strength. Reported values represent the mean ζ-potential obtained from three independent measurements, with corresponding standard deviations.

### Fourier Transform Infrared Spectroscopy (FTIR)

FTIR spectra of the samples were recorded using an Alpha-II compact FT-IR spectrometer (Bruker, Germany). Prior to every measurement, the sample stage was cleaned with 70% ethanol to eliminate any contamination. About 20 μL of each sample was placed directly onto the sample stage. Each spectrum was collected by cumulating 32 background and 32 sample scans with a resolution of 4 cm^–1^ and a wavenumber range of 4000–400 cm^–1^.

### Thermogravimetric Analysis (TGA)

The presence of macromolecular crowders in the coacervate phase was also investigated via thermogravimetric analysis (TGA). The coacervate, supernatant, and buffer samples were examined using a thermogravimetric analyzer (TA Instruments, USA). Approximately 20 mg of each sample was heated in an aluminum pan from 25 to 550 °C at a rate of 10 °C per minute. Differential thermogravimetric (DTG) plots of the data were used to better locate the unique melting or degradation temperatures of the crowder molecule.

### Microscopic Appearance

Complex coacervate droplets were visualized using an optical microscope (Panthera C2, Motic Microscopes, USA). Approximately 30  μL of the coacervate sample was carefully collected and placed onto a concavity slide, which was then covered with a coverslip. Optical images were captured using bright-field (BF) mode. To determine droplet size distributions, images were acquired at multiple regions across each sample, and droplet diameters were measured using ImageJ software.^41^ For each condition, a minimum of 100 droplets were measured and analyzed to ensure statistical robustness.

## Results and Discussion

### Crowding Leads to an Early Onset of Complex Coacervation

In this study, BSA was titrated into PDADMAC solutions, and the turbidity of each sample was measured to track phase separation. The initial concentrations of BSA and PDADMAC used for the titrations were 100 and 2 mg/mL, respectively, in all solution conditions. Figure 1a shows the turbidity as a function of the BSA/PDADMAC molar ratio (*r*) under three solution conditions. The *r* value at which turbidity reached 30% was used to indicate the onset of coacervation. As shown in Figure 1a, visible turbidity began to increase at approximately 17 in NaCl, 7 in Ficoll, and 1 in PEG-crowded conditions. These results reveal an early onset of coacervation in the presence of macromolecular crowders, and similar results were also observed in other published work.^42,43^ To rationalize this behavior, we initially thought that crowding could lead to an increased effective concentration of biomacromolecules, which subsequently enhanced intermolecular encounters and promoted earlier coacervation.

**Figure 1:**
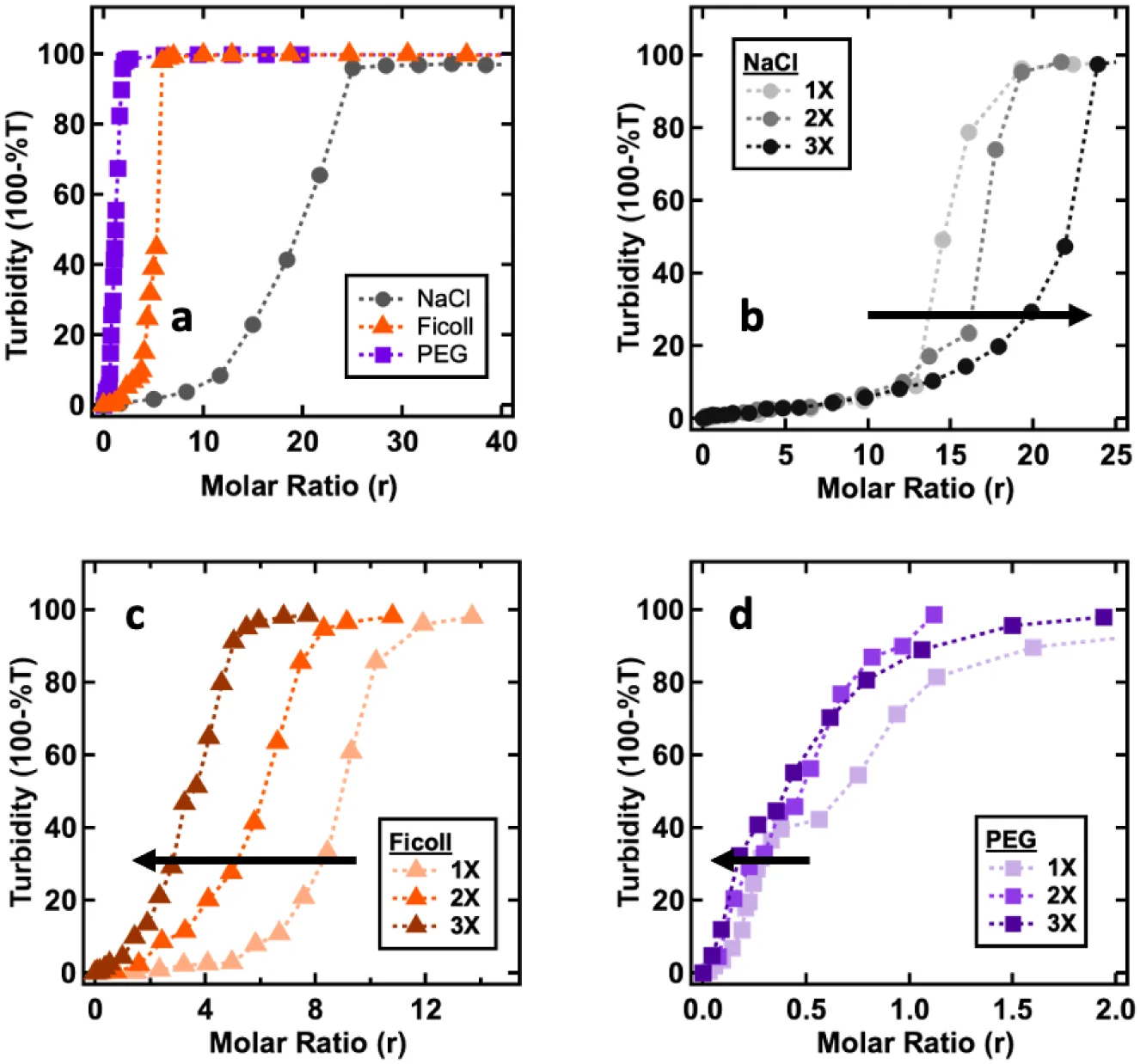
(a) Turbidimetric titrations of BSA into PDADMAC conducted in NaCl, Ficoll, and PEG solution environments. (b-d) Turbidimetric titration results obtained at systematically increased BSA and PDADMAC concentrations under each solution condition, where 1× denotes the lowest and 3× the highest total macromolecular concentration. BSA concentrations were 50, 100, and 150 mg/mL, with corresponding PDADMAC concentrations of 2, 4, and 6 mg/mL for the 1×, 2×, and 3× systems, respectively. The arrows in panels (b-d) indicate the shift in the onset of complex coacervation with increasing BSA and PDADMAC concentrations under different solution conditions.

To test this hypothesis, we performed turbidimetric titrations at systematically higher BSA and PDADMAC concentrations in both dilute and crowded conditions (Figure 1b-d). If volume exclusion were the sole mechanism, then increasing BSA and PDADMAC concentrations in dilute solution should reproduce the same early-onset effect. However, under dilute conditions, increasing concentrations, in fact, delayed the onset of coacervation, indicating that even more BSA was required to initiate phase separation. Under crowded conditions, the same increase in concentration led to an earlier onset. The contrasting trends between dilute and crowded conditions suggest that the early onset cannot be attributed to increased effective concentration alone; macromolecular crowding must contribute through additional mechanisms beyond simple volume exclusion.

### Effects of Crowding on Macromolecular Conformation Prior to Complex Coacervation

To better understand the molecular mechanism underlying the phase separation of BSA and PDADMAC in the presence of crowders, we next examined the conformations of BSA and PDADMAC individually under crowded conditions. If volume exclusion alone is insufficient to explain the early onset of coacervation, then potential changes in the conformation of either BSA or PDADMAC are worth looking into. To evaluate this possibility, we first measured the conformation of BSA in both dilute and crowded solution conditions using an intrinsic fluorescence assay (Figure 2). As shown in Figure 2a, the fluorescence emission spectra of BSA collected under all three solution conditions were almost identical, indicating that the conformation of BSA did not change in the presence of crowders.

**Figure 2:**
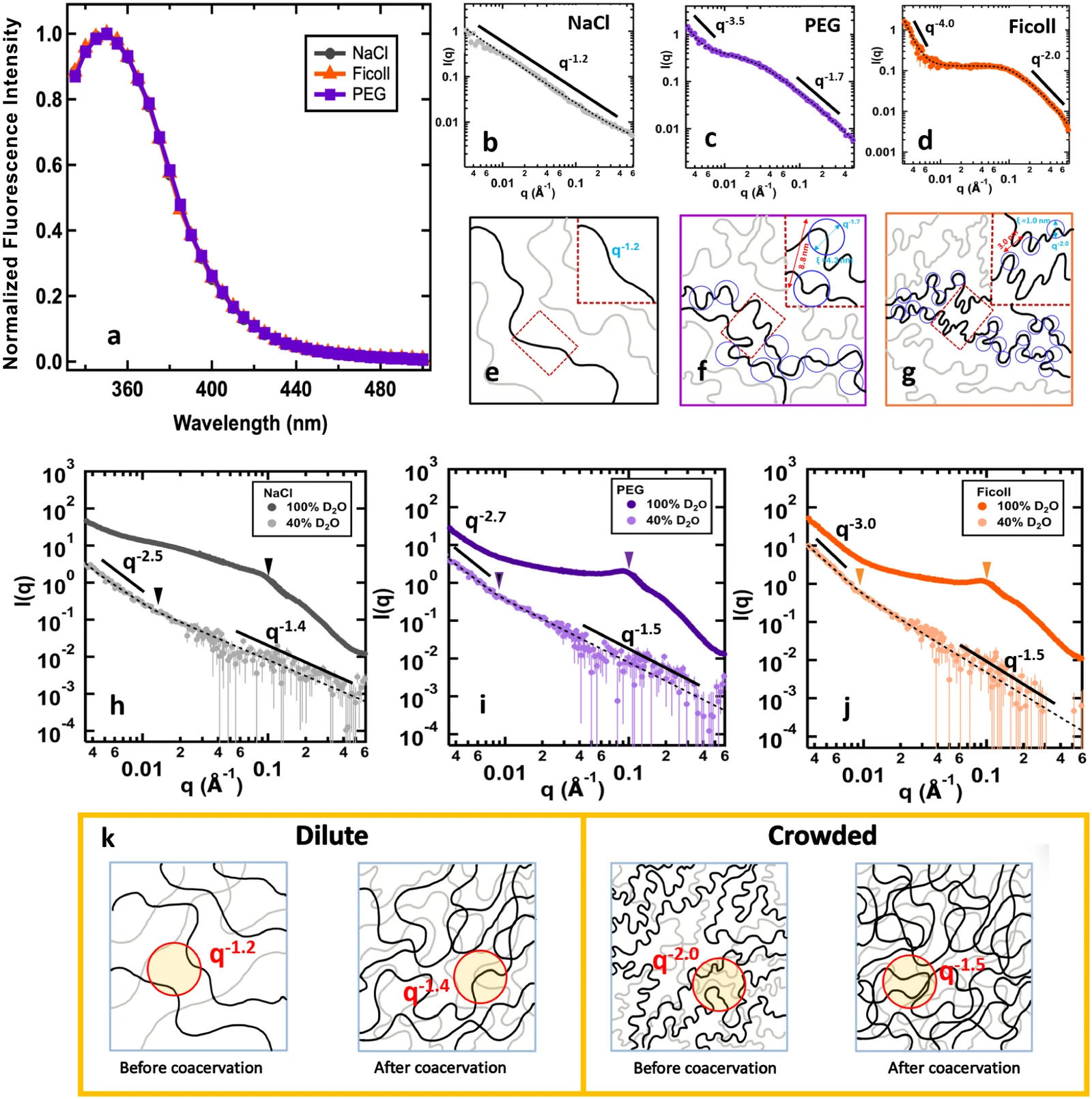
(a) Fluorescence emission spectra of BSA in NaCl, Ficoll, and PEG solution conditions. (b) SANS profile of 6 mg/mL PDADMAC in NaCl solution. (c-d) SANS profiles of PDADMAC prepared at effective concentrations corresponding to those calculated under PEG- and Ficoll-crowded conditions. In these scattering profiles, dotted lines represent fits to the experimental data. (e-g) Schematic illustrations of the possible PDADMAC conformations in different environments. (h-j) SANS scattering profiles of BSA-PDADMAC complex coacervates prepared under no-crowding (h), PEG-crowded (i), and Ficoll-crowded (j) conditions, measured in 100% D_2_O and 40% D_2_O solutions. (k) Schematic illustration of PDADMAC conformations under dilute and crowded conditions before and after complex coacervate formation.

Next, the conformation of PDADMAC was examined in different solution environments using small-angle neutron scattering (SANS). SANS measurements were first performed on 6 mg/mL (0.0132 μM) PDADMAC solutions prepared in dilute condition (NaCl). Under dilute condition, the scattering profile of PDADMAC was fitted using a power-law model. The *q*-exponent was fitted to 1.2, indicating that PDADMAC exhibited a rigid chain conformation (Figure 2b) within the measurable length scale. We also measured 6 mg/mL PDADMAC prepared in PEG- and Ficoll-crowded conditions to investigate the effect of macromolecular crowding on PDADMAC chain conformation. However, extracting the scattering signal from PDADMAC alone was difficult for two reasons. First, the crowders were present at high concentrations (60 mg/mL PEG and 210 mg/mL Ficoll), meaning their scattering strongly dominated the total signal. Second, because the crowder molecules contain hydrogen, they introduced a large incoherent background in the SANS profiles, which further buried the PDADMAC contribution in the high-*q* region, masking the form factor coming from the polymer alone. To get around this problem, we considered that crowder molecules physically occupy a large fraction of the solution volume, which means that the polymer chains are confined to a smaller accessible volume and the local PDADMAC concentration is effectively increased. Using excluded-volume calculations, we estimated that Ficoll occupied roughly 80% of the solution volume, while PEG occupied about 45% of the total volume. Based on these volume fractions, we calculated the effective concentration of PDADMAC to be 12 and 35 mg/mL under PEG- and Ficoll-crowded conditions, respectively (see calculations in Supporting Information).

We then performed SANS measurements on PDADMAC samples prepared at 12 and 35 mg/mL without the crowder (Figure 2c and d). Unlike the 6 mg/mL PDADMAC sample, these scattering profiles were best described by a combined model consisting of a correlation-length term and a hard-sphere structure factor. This model reflects a transition in which PDADMAC chains no longer behave as extended chains but instead form an interconnected network characterized by an average mesh size, ξ, also known as the correlation length.^44,45^ This mesh size is commonly described in terms of polymer “blobs” and represents the local packing of charged segments.^45^ The hard-sphere structure factor accounts for repulsive interactions among these blob-like domains and enables estimation of the average inter-blob spacing. For PDADMAC prepared at the effective concentration corresponding to PEG-crowding conditions, the high-*q* exponent (*m*) was fitted to 1.7, consistent with a flexible chain conformation. The correlation length, ξ, was 4.3 nm, corresponding to the characteristic size of the blob structure, while the average distance between blobs was 8.8 nm. In the low-*q* region, the scattering profiles exhibited a slope of 3.5, indicating that the PDADMAC chains formed a highly overlapping network structure.

Similarly, the scattering profile measured for PDADMAC prepared at an effective concentration corresponding to Ficoll crowding conditions (Figure 2d) was also fitted using the combined model. The high-*q* exponent was fitted to 2.0, indicating that PDADMAC chains adopted Gaussian coil conformations.^46^ The correlation length was fitted to 1.0 nm, with the average distance between blobs was fitted to 3.0 nm. Both of these values were smaller than those obtained under PEG conditions, suggesting that Ficoll promoted a more coiled and constrained PDADMAC conformation and brought the chains into closer proximity, as reflected by the reduced inter-blob spacing. In addition, the low-*q* region showed a slope of 4, suggesting the formation of larger structures, similar to that observed in PEG. Collectively, the analysis of the scattering profiles measured from PDADMAC at different concentrations suggested that increased effective concentration arising from macromolecular crowding forced PDADMAC chains into more coiled conformations. This brought neighboring chains into close proximity despite their electrostatic repulsion, subsequently promoting their aggregation into large networks.

### Effects of Crowding on Macromolecular Conformation upon Complex Coacervation

At this point, we know that the conformation of BSA remained unchanged, with or without crowders, whereas PDADMAC transitioned from a rigid chain to a coiled conformation that further overlapped into network structures. We next used SANS to investigate how the conformation and assembly of BSA and PDADMAC molecules evolved during phase separation in different crowded environments. To this end, BSA-PDADMAC coacervates were prepared in two solvent contrasts: 100% D_2_O and 40% D_2_O. In 100% D_2_O, both BSA and PDADMAC contributed to the measured neutron scattering, whereas in 40% D_2_O, the scattering length density of the solvent matched that of BSA, making the protein invisible to neutrons. As a result, the measured scattering in 40% D_2_O arose solely from PDADMAC within the complex coacervates, enabling *in situ* selective observation without the need for labeling.

SANS profiles of BSA-PDADMAC complex coacervates prepared in both 100% D_2_O and 40% D_2_O are shown in Figure 2h-j. In general, the scattering profiles measured from coacervates prepared in 100% D_2_O all exhibited a pronounced shoulder in the *q*-range of 0.08–0.1 Å^–1^, corresponding to a characteristic length scale of approximately 6–8 nm. It is anticipated that this feature arose due to the center-to-center distance between adjacent BSA molecules sequestered within the dense coacervate phase.^47^ To further examine differences in BSA packing under various crowding conditions, the scattering data were presented in Kratky plots (Figure S4 in the Supporting Information), which allowed the peak positions among different scattering profiles to look more distinct and easier to compare. Our results show that the BSA molecules were most closely packed in coacervates formed in the presence of Ficoll, with an average inter-protein distance of approximately 6.1 nm, followed by PEG at 6.3 nm, while coacervates formed under dilute conditions exhibited a larger average spacing of approximately 6.9 nm. In addition to shifts in peak position, differences in peak shape were also observed across the three conditions. The correlation peak for coacervates prepared in NaCl was broader, reflecting a wider distribution of protein–protein distances, whereas those observed under PEG- and Ficoll-crowded conditions were sharper and better defined, indicating that proteins were more uniformly spaced within the coacervate phase. Together, these observations reveal tighter and more uniform BSA packing in coacervates formed in the presence of PEG and Ficoll. Additional differences among the three conditions were observed in the intermediate to low-*q* region of the scattering profiles. Coacervates prepared in NaCl showed a gradual increase in scattering intensity toward the low-*q* region, suggesting the formation of large structures. In comparison, coacervates prepared in PEG- and Ficoll-crowded environments exhibited a plateau region over the *q*-range (0.01–0.08 Å^–1^). The appearance of this plateau suggests that, over this length scale, the scattering intensity was strongly influenced by repulsions among closely packed BSA molecules. As *q* decreased further, an upturn in scattering intensity became evident, indicating the formation of larger BSA-PDADMAC assemblies.

The scattering profiles measured in 40% D_2_O contrast looked significantly different from those obtained in 100% D_2_O (Figure 2h-j). The most notable difference was the disappearance of the intermediate-*q* shoulder that was clearly observed in the fully deuterated condition. As discussed earlier, this shoulder was attributed to the close packing of adjacent BSA molecules within the coacervate phase. In 40% D_2_O, the scattering length density of the solvent matched that of BSA, making the protein effectively invisible to neutrons. As a result, such BSA-related features were absent from the scattering profiles. Aside from the loss of the intermediate-*q* shoulder, the scattering profiles of coacervates prepared in 40% D_2_O were comparable across all three crowding conditions and exhibited two distinct power-law regimes (Figure 2h-j). In the high-*q* region, the scattering profiles displayed power-law exponents ranging from 1.4 to 1.5 for all conditions examined. In the 40% D_2_O solution, neutron scattering came solely from PDADMAC chains, and such an exponent suggested that PDADMAC chains adopted a flexible chain conformation within the coacervate phase, regardless of the crowding environment. In the low-*q* region, the scattering profiles measured from PDADMAC in 40% D_2_O exhibited a pronounced upturn with power-law exponents from 2.5 to 3.0, suggesting that PDADMAC chains entangle into larger network structures and revealing that the internal architecture of the coacervate was underpinned by an entangled PDADMAC network.

Our studies have shown that the conformation of PDADMAC prior to coacervate formation depends strongly on solution conditions. In dilute NaCl solution, PDADMAC chains exhibited a relatively rigid conformation within the measured *q* range due to electrostatic repulsions along the charged backbone (Figure 2l). Upon incorporation into the complex coacervate, the high-*q* exponent increased slightly to 1.4, suggesting a transition toward a more flexible chain conformation. This change was likely due to the neutralization of backbone charges upon association with oppositely charged BSA molecules. Under crowded conditions, excluded-volume effects forced PDADMAC chains into coiled conformations and brought them into close proximity, promoting their entanglement into heavily overlapping network structures prior to coacervation. Upon coacervate formation, however, these chains relaxed to a more extended conformation similar to that observed in coacervates formed under dilute conditions. Notably, the shoulder feature associated with correlation-length effects observed prior to coacervation was absent after coacervate formation, suggesting that local chain packing became less constrained within the coacervate phase. Collectively, our results suggest that PDADMAC underwent conformational changes upon coacervate formation that differed depending on whether crowders were present. In dilute conditions, PDADMAC became more flexible upon binding to BSA, whereas in crowded environments, PDADMAC chains became more relaxed from a compressed conformation (Figure 2L).

In dilute solutions, charged polymers are likely to adopt expanded conformations due to repulsions along the charged polymer backbone. When in crowded environments, the polymers will experience a reduction in configurational entropy due to excluded-volume effects imposed by surrounding macromolecules.^48^ As crowding increases, spatial confinement promotes chain compaction, incurring an entropic free-energy penalty associated with polymer confinement.^49,50^ Based on these considerations, we think PDADMAC in crowded environments can be viewed as adopting a constrained, or “tense,” conformational state characterized by suppressed configurational entropy and elevated free energy. This picture is supported by our SANS results, which exhibited significant differences in the scattering profiles of PDADMAC prepared at effective concentrations compared to those measured under dilute conditions, consistent with the anticipated crowding-induced conformational changes. Upon introduction of oppositely charged BSA, this unfavorable constraint was relieved through electrostatic complexation, accompanied by the release of hydration water and condensed counterions.^51,52^ Binding of the protein locally neutralized polymer charges, and the counterions released into the bulk solution provided a significant entropic driving force for complex formation.^53,54^ The resulting reduction in the electrostatic repulsive interaction along the polymer backbone enabled PDADMAC to relax into a more flexible and extended conformation within the coacervate phase. Intuitively, this behavior is analogous to entropic elasticity in polymer physics. A polymer chain that deforms away from its equilibrium random-coil conformation, either by extension or compression, would result in a loss of configurational entropy and a concomitant free-energy penalty. Classic polymer statistical mechanics describes the elastic response of flexible chains as being dominated by entropic contributions, with the equilibrium coil corresponding to the state of maximum accessible configurations.^55,56^ Single-molecule force–extension experiments further demonstrate that mechanical stretching of a polymer requires work to reduce its conformational entropy, and the removal of the applied force leads to spontaneous relaxation toward the equilibrium state.^56–58^ Similarly, the compression of polymer chains due to confinement effects also restricts accessible configurations and is therefore entropically unfavorable, generating a restoring tendency toward a more relaxed and thermodynamically favorable conformation. In this context, complex coacervation provided a pathway by which PDADMAC chains initially constrained by macromolecular crowding can lower their free energy through electrostatically driven association with BSA, thereby facilitating conformational relaxation and promoting phase separation at lower protein–polymer ratios.

### Structural Basis for the Early Onset of Complex Coacervation in Crowded Environments

The central question motivating this work is “why does complex coacervation occur at lower BSA/PDADMAC ratios in crowded environments?” To address this question, it is helpful to first consider the molecular mechanism of complex coacervation in dilute solutions. Numerous studies, including our own, have shown that coacervation can be induced by changing the protein-to-polymer stoichiometry, most commonly by titrating protein into a polymer solution. At the beginning of the titration, a small number of protein molecules bind electrostatically to polymer chains, forming intra-polymer complexes that can be described as protein-decorated polymers. As the concentration of protein is increased, more binding sites along the polymer backbone become occupied, progressively reducing the net charge of intra-polymer complexes. When the complexes approach charge neutrality, electrostatic repulsion is sufficiently reduced to allow aggregation, leading to the formation of inter-polymer complexes^59^ and, ultimately, the highly overlapping protein–polymer coacervate network.

To examine whether this mechanism applies to the present system, we performed BSA-to-PDADMAC titrations in different crowded conditions while monitoring both (Figure 3a-c) turbidity and zeta potential (ζ-potential). Under dilute conditions, complex coacervation began when the *r*-value reached 12. At this ratio, the ζ-potential of the BSA-PDADMAC complexes decreased substantially, indicating a significant reduction in net charge. Together, these results suggest that coacervation under dilute conditions was initiated near charge neutrality, driven by reduced electrostatic repulsion between complexes. This behavior is consistent with the widely accepted view that charge neutralization plays a key role in complex coacervation.^60^ In addition, turbidimetric titrations performed at higher protein and polymer concentrations (Figure S5) exhibited similar trends, further supporting the proposed molecular mechanism underlying complex coacervation. It is therefore evident that complex coacervation under dilute conditions requires the formation of charge-neutral BSA-PDADMAC complexes. This mechanism can now explain the delayed onset of complex coacervation observed under dilute conditions at higher polymer concentrations (Figure 1b): as more PDADMAC was added, more BSA molecules were required to form charge-neutral BSA-PDADMAC complexes, increasing the BSA-PDADMAC ratio needed for coacervation and thereby delaying its onset. In addition, at higher PDADMAC concentrations, the corresponding increase in counterion concentration may further weaken the electrostatic interactions between BSA and PDADMAC through charge screening, requiring additional BSA molecules to compensate for this weaker interaction in order to reach charge neutrality. Both effects would act in the same direction, contributing to the increased BSA-PDADMAC molar ratio needed for coacervation and the resulting delayed onset observed at higher polymer concentrations.

**Figure 3:**
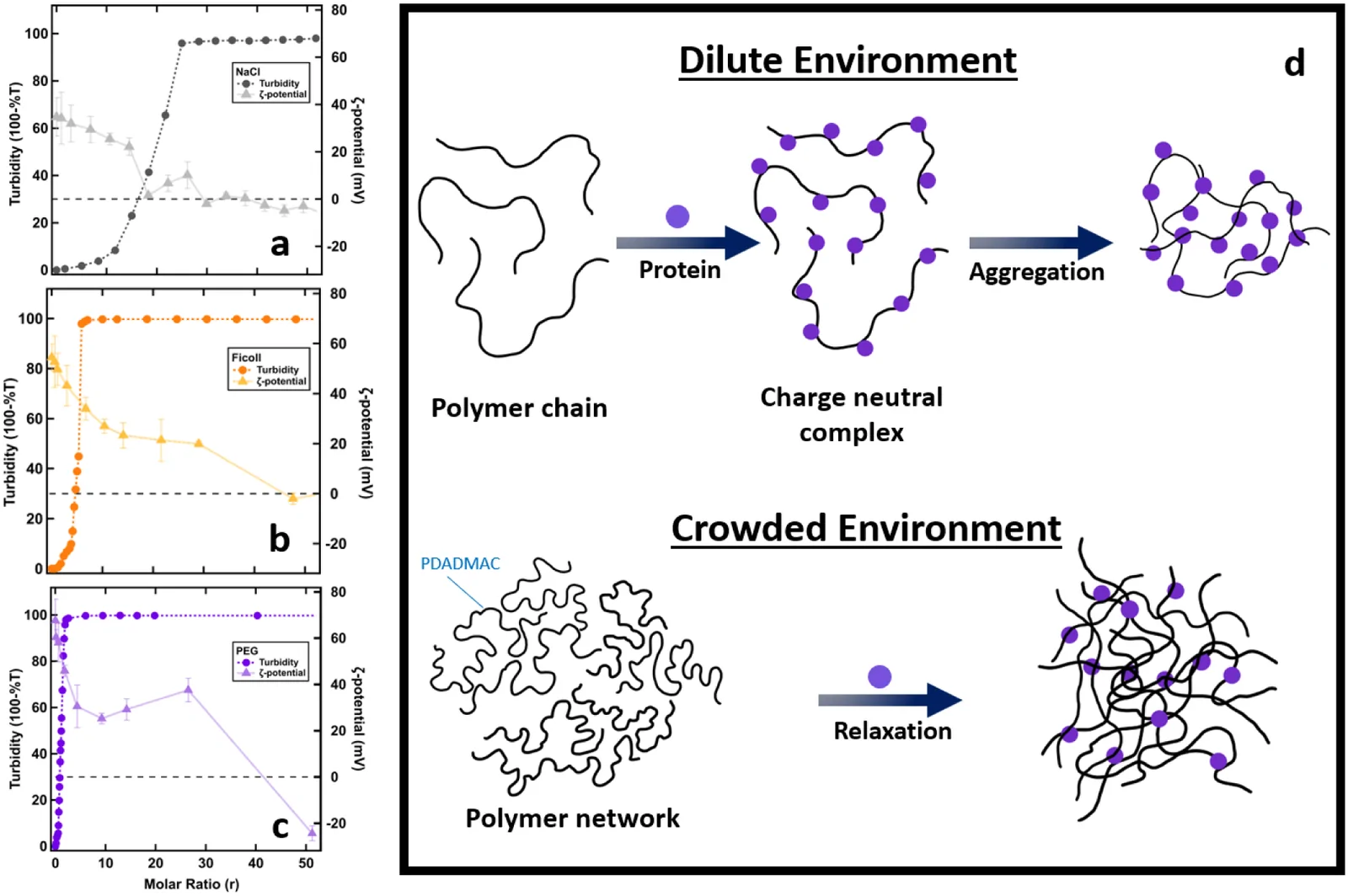
(a-c) Turbidimetric titration and ζ-potential measurements obtained from BSA-to-PDADMAC titrations performed in different conditions. The concentrations of BSA and PDADMAC were 2 and 50 mg/mL, respectively. (d) Schematic illustration of the proposed molecular mechanism underlying complex coacervate formation under dilute and crowded environments. Ficoll or PEG crowding increases the effective concentration of PDADMAC, driving the polymer to adopt a coiled conformation and form a network. Crowder molecules and salt ions are omitted on purpose from the illustration to keep the focus on the protein and polymer.

A key difference between dilute and crowded conditions is revealed by our SANS measurements. In crowded solutions (with the presence of Ficoll or PEG), where the effective concentration of PDADMAC was much higher, PDADMAC chains were found to form closely packed, interconnected structures even before the addition of BSA. In this preorganized state, polymer chains already resided in close spatial proximity. As a result, the introduction of BSA not only provided favorable electrostatic interactions but also allowed complexes to form more readily because neighboring PDADMAC chains were already positioned near one another. In this way, the structural preorganization lowered the barrier for inter-chain association and network formation, enabling coacervation to occur at a lower protein–polymer ratio. Consistent with this mechanism, the ζ-potential of the coacervates formed under crowded conditions remained distinctly nonzero at the point of coacervation onset (Figure 3b, c), in contrast to the near-neutral ζ-potential observed under dilute conditions. This suggests that, under crowding, coacervation does not require the BSA-PDADMAC complexes to first reach charge neutrality. The preorganized PDADMAC network lowers the barrier for association independently of full charge neutralization, allowing coacervation to proceed while a net charge is still retained on the resulting complexes. The schematic in Figure 3d provides a side-by-side comparison of the molecular mechanisms governing complex coacervation under dilute and crowded conditions.

### Macroscopic Properties of Coacervates Prepared under Different Solution Environments

From the SANS results, we know that the conformation of PDADMAC differed between crowded and dilute environments. However, after forming coacervates with BSA, the resulting BSA-PDADMAC coacervate structures appeared similar regardless of the crowding conditions under which they were formed. To gain further insight into coacervates prepared under different conditions, we next examined several macroscopic properties of the coacervate phase. We first quantified the concentrations of BSA and PDADMAC in coacervates prepared under different conditions. Under dilute conditions, the concentrations of BSA and PDADMAC in the coacervate phase were 160 ± 13 mg/mL and 29 ± 4 mg/mL, respectively. In the presence of macromolecular crowders, the BSA concentration increased significantly, reaching 290 ± 18 mg/mL in Ficoll-containing systems and 275 ± 24 mg/mL in PEG-containing systems. The PDADMAC concentration also increased in crowded environments, with the highest value observed in the PEG system (45 ± 5 mg/mL). Although macromolecular crowding led to changes in coacervate composition relative to dilute conditions, the compositional differences between Ficoll- and PEG-crowded systems were not statistically significant (Figure 4a).

**Figure 4:**
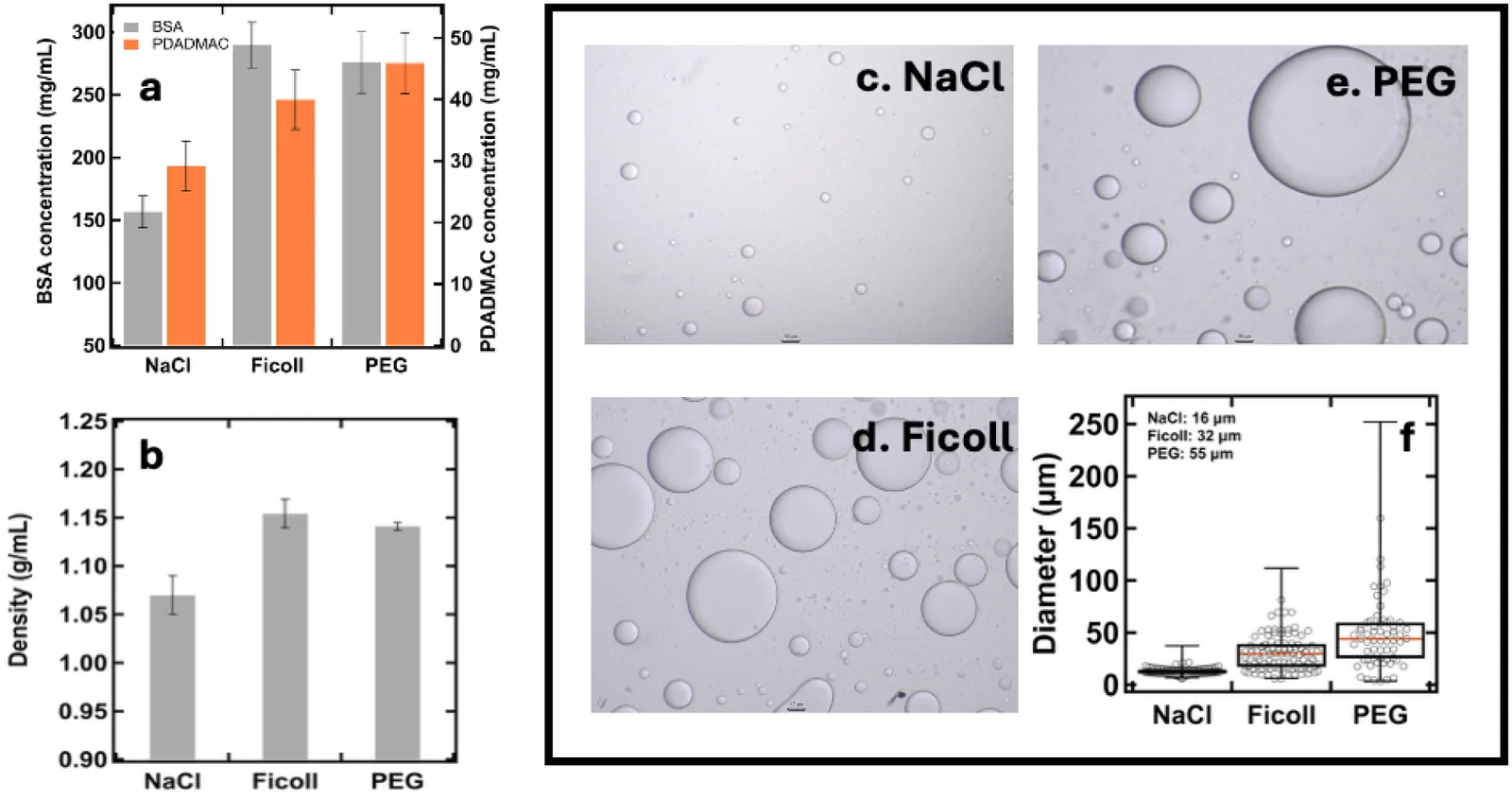
(a) Concentration of BSA and PDADMAC in the coacervate phase. (b) Density of the coacervate phase. Error bars correspond to one standard deviation from three repeated measurements. (c-e) Microscopic images of coacervate samples prepared in different solution environments. Scale bars represent 10 μm. (f) Box plots showing the size distribution of coacervate droplets formed under the three solution conditions at a molar ratio of 35. The lower and upper boundaries of the box represent the 25th and 75th percentiles, respectively. The lower and upper limits represent the minimum and maximum values, respectively, and the median is represented by the horizontal line within the box.

We also measured the density of the coacervates and found that coacervates formed in the presence of crowders exhibited higher densities than those prepared under dilute conditions (Figure 4b). To determine whether this increase arose from the incorporation of crowders into the dense phase, we examined the partitioning of PEG and Ficoll into the coacervate and supernatant phases. To this end, Fourier transform infrared spectroscopy (FTIR) and thermogravimetric analysis (TGA) were performed on both phases to assess the distribution of the crowders. The FTIR spectrum collected from Ficoll buffer (Figure S6 in the Supporting Information) showed two characteristic peaks at 1055 and 1001 cm^–1^, attributing to the C–O–C stretching vibrations of sucrose units within the backbone of Ficoll.^61^ These peaks were clearly present in the supernatant but absent in the coacervate phase, indicating that Ficoll did not partition into the coacervate phase. Similarly, PEG-specific FTIR features were detected only in the supernatant and not in the coacervate phase, consistent with our previous observations.^47^

TGA analysis provided complementary evidence for crowder partitioning. The derivative thermogram (DTG) obtained from the TGA spectrum measured for the Ficoll solution exhibited a degradation maxima peak around 250 °C, corresponding to the thermal decomposition of sucrose units (Figure S6 in the Supporting Information).^62^ This peak was observed in both the Ficoll solution and the supernatant but not in the coacervate phase, confirming that Ficoll was excluded from the coacervate phase. The TGA spectrum measured for the PEG solution showed a shallow decomposition peak near 390 °C, which was characteristic of PEG thermal degradation.^63–65^ Such feature was found in the PEG solution as well as the supernatant from the separated sample, whereas there was no sign of PEG decomposition in the coacervate phase. Such partitioning behavior differs from previously published results on protein–polymer coacervate systems,^66^ highlighting that crowder partitioning is dependent on the specific macromolecular components forming the coacervate. It is also worth noting that, although the supernatant is enriched with crowders, it still contains fractions of BSA and PDADMAC molecules that are not incorporated into the dense phase, and the amount remaining in the supernatant is dependent on the crowder used. Lastly, rheological measurements confirmed liquid-like behavior of coacervates formed under all three conditions (Figure S7 in the Supporting Information), although those formed under crowded conditions exhibited enhanced complex viscosity and frequency dependence, suggesting increased structural connectivity. These similarities in viscoelastic behavior were consistent with the SANS results, which revealed that the coacervates shared similar structures at the nano- and mesoscale, regardless of the solution conditions.

### Effects of Crowders on Droplet Coalescence

One of the most apparent features of complex coacervates is the droplet morphology and size distribution when observed under the microscope. To examine how solution conditions influence these properties, we compared the appearance of coacervate droplets formed under three different conditions. In general, coacervates formed in the presence of crowders exhibited larger droplets with broader size distributions than those formed under dilute conditions, with PEG producing greater heterogeneity than Ficoll (Figure 5). We also looked at the growth of coacervate droplets upon mixing. It was found that under dilute conditions, BSA-PDADMAC complexes initially formed small, relatively uniform coacervate droplets that gradually coalesced into larger droplets over time. This behavior is consistent with classical droplet coarsening, whereby coacervate droplets grow progressively through collision and merging events, as has been reported for polyelectrolyte coacervate systems formed under dilute conditions.^67,68^ In crowded environments, droplet coalescence occurred more rapidly and extensively (Figure S8 in the Supporting Information), ultimately giving rise to the broader and more heterogeneous size distributions observed under these conditions.

**Figure 5:**
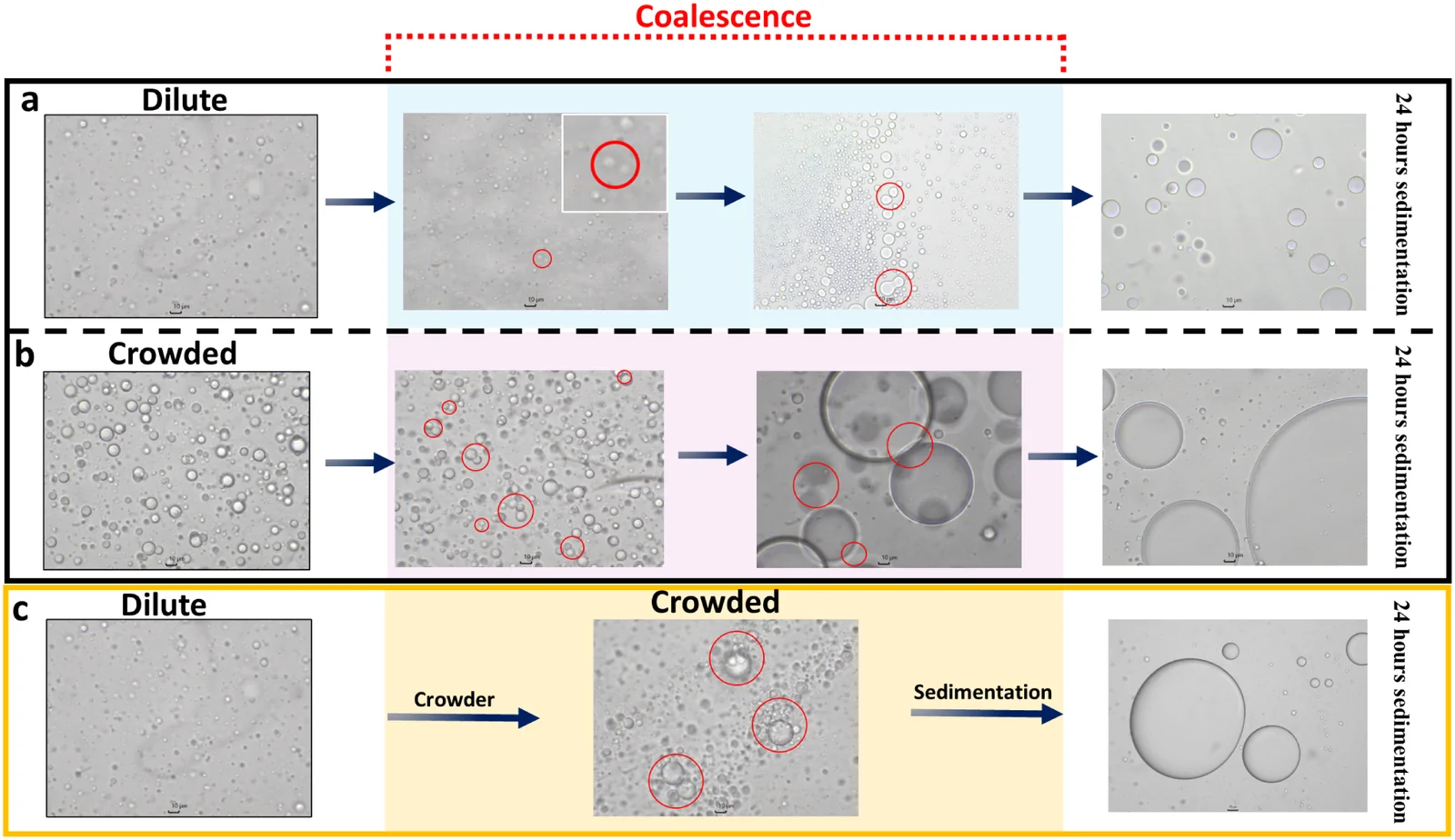
(a) Under dilute conditions, mixing BSA and PDADMAC produced small droplets with narrow size distributions that increased slightly over time due to coalescence (inset). (b) Under crowded conditions, frequent coalescence of smaller droplets led to the formation of larger droplets with broader size distributions. (c) When crowder was added to coacervates previously prepared under dilute conditions, neighboring droplets were driven to coalesce into larger droplets, yielding a morphology similar to that observed under crowded conditions (as shown in panel b).

To investigate the origin of the enhanced coalescence observed in crowded environments, we performed a series of supernatant-exchange experiments. When the supernatant of phase-separated BSA-PDADMAC coacervates formed in NaCl solution was replaced with PEG solution, without disturbing the dense phase, the relatively uniform droplets rapidly merged together (Figure S9 in the Supporting Information), forming larger and more heterogeneous droplets that closely resembled coacervates formed directly under crowded conditions after 24 h of sedimentation (Figure 5b). Conversely, replacing the supernatant of coacervates formed under PEG-crowded conditions with NaCl solution led to reduced droplet size (Figure S9 in the Supporting Information). Collectively, our results demonstrate that although crowders were excluded from the dense coacervate phase, their presence in the coexisting supernatant phase has a direct impact on the droplet size distribution within the dense phase. This finding is significant because it establishes that the coacervate system, despite being phase-separated, remains highly dynamic, with the two coexisting phases remaining in active communication.

## Conclusion

Our study demonstrates that complex coacervation proceeds through distinct pathways under dilute and crowded conditions. In dilute solution, coacervation is driven by charge neutralization and the gradual assembly of protein–polymer complexes. In crowded environments, excluded-volume effects drive polymers into coiled conformations and preorganize them into networks, priming the system for near-instantaneous coacervation without the strict requirement for charge neutrality. Despite these mechanistic differences, crowders are found to be excluded from the coacervate phase upon phase separation, and scattering results reveal that coacervates formed under both conditions share comparable mesoscopic structures. Notably, coacervates formed under crowded conditions incorporate more protein and polymer and exhibit denser internal networks. Our results also show that the presence of crowders in the supernatant, despite being excluded from the dense phase, promotes droplet coalescence, giving rise to larger droplets with broader size distributions. This finding highlights that the supernatant and dense phases, although physically separated, remain in active communication and continue to influence each other after phase separation. Collectively, these findings establish a mechanistic framework in which macromolecular crowding promotes polymer preorganization and enables phase separation across a wider range of conditions than is possible in dilute solution. This work provides fundamental insight into how biomolecular condensates can assemble readily and adaptively in a crowded cellular environment.

## Supplementary Material



## Notes

Characterization of Ficoll and PEG using DLS and SAXS, followed by excluded-volume calculation; ITC assessing crowder-protein and crowder-polymer interactions; PDADMAC concentration calibration curves; SANS fitting parameters and Kratky plots for coacervates; turbidimetric titration and ζ-potential measurements of complexes; FTIR and TGA compositional analysis of coacervate, supernatant, and buffer phases; and rheological (frequency-sweep, storage modulus, and loss modulus) characterization of coacervate (PDF)
