# Supplementary material for: Mechanistic Insights into the Formation of Complex Coacervates in Crowded Environments
Source: Biomacromolecules. 2026 Aug 21;27(9):6214–26. doi: 10.1021/acs.biomac.6c01306 (PMC13580021; doi:10.1021/acs.biomac.6c01306)
Supplement: Supplementary file 1 [file bm6c01306_si_001.pdf]

Supporting Information

Mechanistic Insights into the Formation of Complex Coacervates in Crowded Environments

*Shanta Biswas[1], Sai Venkatesh Pingali[2], Katie T Tran[1],Qingqiu Huang[3], Xiaobing Zuo[4], Amy Y. Xu[1]**

[1]Department of Chemistry, Louisiana State University, Baton Rouge, Louisiana 70803, USA
[2]Neutron Scattering Division, Oak Ridge National Laboratory, Oak Ridge, Tennessee 37830, USA
[3]Cornell High Energy Synchrotron Source (CHESS), Cornell University, Ithaca, New York 14853, USA
[4]X-Ray Source Division, Advanced Photon Source, Argonne National Laboratory, Lemont, Illinois 60439, USA.

Size, Shape, and Volume Occupied by Crowder Molecule

The apparent sizes of Ficoll and PEG were measured using a DLS instrument (Litesizer500, Anton Paar, USA) at 20 °C. The instrument was equipped with a single wavelength laser diode emitting light of 658 nm. All solutions were filtered through 0.45 µm syringe filters to remove dust prior to all measurements. The samples were freshly prepared, and multiple measurements were performed for each sample to obtain an average hydrodynamic radius (R_h). The R_h was calculated using the Stokes-Einstein equation-

$$R_h = \frac{k_B T}{6\pi\eta D_T} \qquad \text{Eq. S1}$$

where k_B is the Boltzmann constant, T is the absolute temperature, D_T is the translational diffusion coefficient, and η is solvent viscosity.

Small-Angle X-ray Scattering (SAXS). The SAXS measurement of Ficoll in 50 mM NaCl was conducted on BioSAXS beamline (Sector 7A1) at Cornell High Energy Synchrotron Source

(CHESS), Ithaca, New York, USA using an air-gap set-up. Samples were loaded into a custom-fabricated cell sealed with Kapton film on both sides, with a path length of 2.0 mm and a sample volume of approximately 15 μL. The scattering patterns were collected with an X-ray energy of 11.2577 keV. The scattering measurements covered the q-range from 0.0087 $Å^{-1}$ to 0.44 $Å^{-1}$, which corresponds to a length-scale from 2 nm to 72 nm. The collected SAXS data were reduced using the BioXTAS RAW software[1].PEG solution prepared in 50 mM NaCl was measured at the 12-ID-B beamline at the Advanced Photon Source (APS), Argonne National Laboratory, using 13 keV X-rays over a q-range of 0.003-0.88 $Å^{-1}$. The resulting scattering data was processed to obtain reduced intensity profiles for subsequent analysis. Reduced data collected from CHESS and APS were further analyzed using NCNR analysis macro package built into IgorPro software[2, 3]. The scattering vector q and intensity is defined as similar to it expressed in Eq, S3 and S4.

Ficoll is a high molecular weight (70 kDa) neutral polysaccharide synthesized through copolymerization of sucrose with epichlorohydrin. The high density of hydroxyl groups renders Ficoll highly soluble in aqueous media. As Ficoll lacks ionizable groups, it does not interact electrostatically with charged macromolecules, which makes it an ideal candidate for studying its effects as macromolecular crowder on charged macromolecules [4, 5].

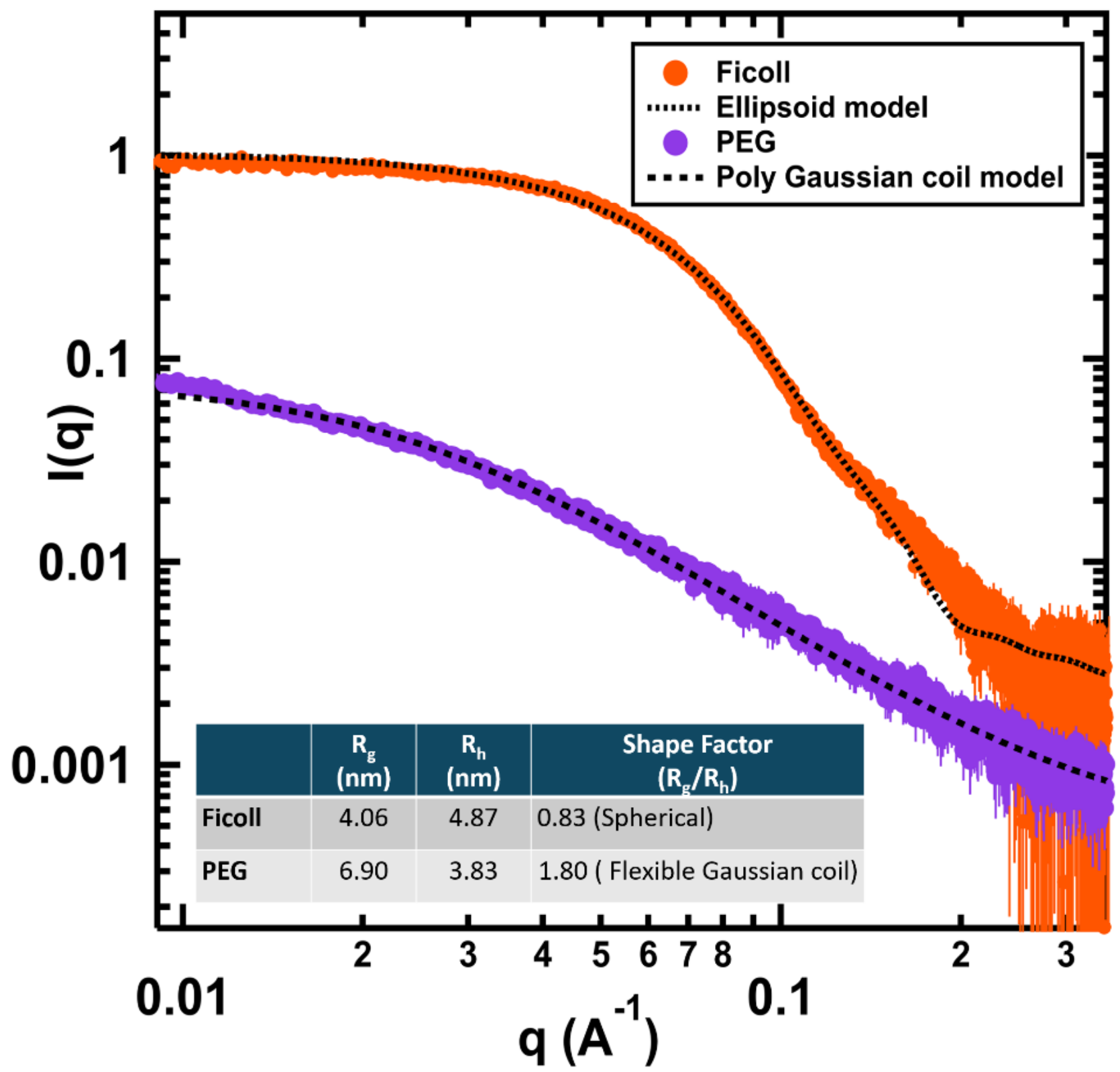

Figure S1. SAXS profiles of Ficoll and PEG in 50 mM NaCl solution. The scattering profiles were fitted using Ellipsoid and Gaussian coil model, respectively to extract the radius of gyration (R_g).

Ficoll can bind water in quantities 2.5 to 3 times greater than its own volume creating a thicker hydration shell surrounding it[6]. This thick hydration shell surrounding Ficoll contributes to its effective size in solution, where water-solute interactions play a pivotal role. Accordingly, the R_h of Ficoll was determined to be 4.87±0.20 nm, which is larger than the radius of gyration (R_g = 4.06 nm) obtained from SAXS. The corresponding shape factor was calculated as 0.83, inferring a shape close to a sphere[7]. On the other hand, Polyethylene glycol (PEG) is a highly water-soluble, inert synthetic polymer widely used as a macromolecular crowding agent[8-10]. Comparing the R_h values, PEG is smaller than that of Ficoll in 50 mM NaCl. The R_g and R_h of PEG were determined independently by DLS, yielding values of 6.90±0.05 nm and 3.83±0.22 nm, respectively. The

resulting shape factor (R_g/R_h = 1.80) of PEG indicates a random coil[11, 12] like (Figure S1) conformation. s

Using the hydrodynamic radius extracted from DLS, the hydrodynamic volume of each crowder molecule was estimated, which subsequently enabled us to calculate total volume occupied by the crowders in solutions. The degree of volume occupancy was then used to determine the effective local concentration of the PDADMAC within the crowded environment. Assuming both Ficoll and PEG adopt approximately spherical conformations in hydrated state; the hydrodynamic volume[13-15] was calculated using the following equation:

$$Volume\ occupied\ by\ crowder\ = \frac{4}{3}\pi R_h^3 N_A \quad \text{Eq. S2}$$

Where, R_h is the hydrodynamic radius (nm) of the crowder, and N_A is the Avogadro's number. The calculated volume occupancy of the crowders was approximately 80% for Ficoll and 45% for PEG. Taking this excluded volume into account, the local effective concentration of PDADMAC initially prepared at 6 mg/mL was estimated to increase to approximately 35 mg/mL in Ficoll and 12 mg/mL in PEG environments.

Isothermal Titration Calorimetry (ITC) to Probe Crowder-Protein and Crowder-Polymer Interaction

ITC measurements were carried out using a Nano ITC calorimeter (TA Instruments, USA). Prior to measurement, BSA and PDADMAC solutions were prepared in 50 mM NaCl and dialyzed against the same buffer using a 5-8 kDa molecular weight cutoff membrane to ensure buffer matching. For each experiment, the sample cell was loaded with either BSA or PDADMAC solution, while the syringe contained either the crowder solution or buffer. Each titration consisted

of 15 injections of 2.01 μL, in which crowder or buffer from the syringe was incrementally added to the BSA or PDADMAC solution. A spacing of 180 seconds between injections was used to allow the system to return to thermal equilibrium. All measurements were conducted at 20 °C with a stirring rate of 300 rpm. To account for heat of dilution, control titrations were performed under identical conditions, and the corresponding heats were subtracted from the raw data. The resulting thermograms were processed and analyzed using NanoAnalyzer software (TA Instruments).

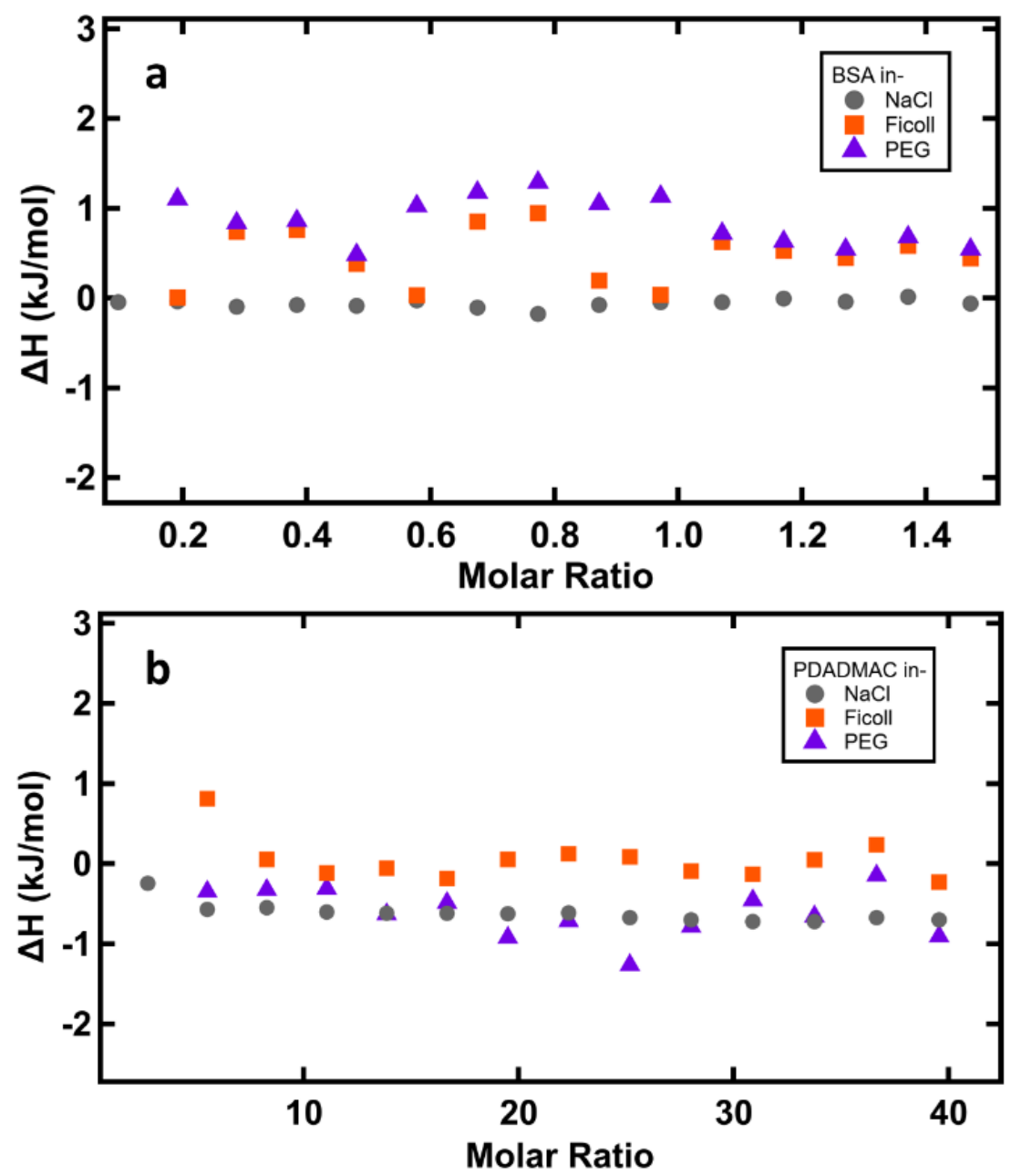

Figure S2. ITC profiles of (a) BSA–crowder and (b) PDADMAC–crowder interactions. Corresponding BSA–buffer and PDADMAC–buffer controls are shown as NaCl in both figures.

The results indicate no significant interaction between the crowder and either BSA or PDADMAC prior to coacervate formation. Therefore, the only contributor to the major interaction is the electrostatic association between BSA and PDADMAC, consistent with complex coacervate formation mechanisms across all solution conditions.

Calibration Plots for PDADMAC Concentration Determination

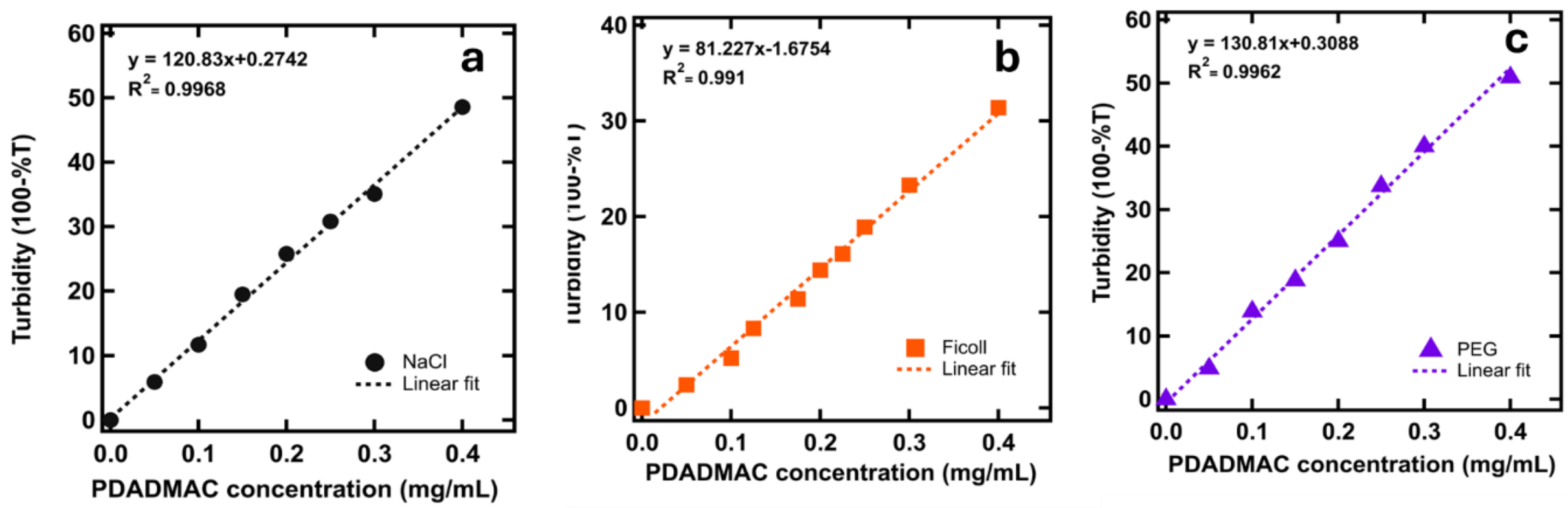

Figure S3. Calibration plots of PDADMAC in (a) NaCl, (b) Ficoll, and (c) PEG solution conditions. The data was fitted to a line. The fitted line equation and the R^2 value are shown on the left side of each figure. These calibration plots were used to determine the PDADAMC concentration in the supernatant for the respective buffer conditions.

Small-Angle Neutron Scattering (SANS)

Table S1. Parameters obtained from SANS fitting

Crowding Condition	Low-q exp.	High-q exp.	Crossover point, q (Å^{-1}), and distance (d=2π/q, nm)	Size of polymer blob, ξ (nm)	Distance between neighboring polymer blobs (nm)	Fitting Model
**PDADMAC in 100% D_2O**						
**NaCl**	1.2 ± 0.1		–	–	–	Power-law Functions
**Ficoll**	4.0	2.0 ± 0.02 (1.98–2.02)	–	1.0 ± 0.01 (0.99–1.01)	3.0 +0.3/-0.17 (2.83–3.3)	Correlation Length/Hard sphere
**PEG**	3.5 ± 0.1 (3.4–3.6)	1.7 +± 0.05 (1.65–1.75)	–	4.3 +± 0.01 (4.29–4.31)	8.8 ± 0.1 8.7-.8.9)	Correlation Length/Hard sphere
**Coacervate in 40% D_2O**						
**NaCl**	2.5 ± 0.2 (2.3–2.7)	1.4 +0.2/–0.1 (1.3–1.6)	0.012 ± 0.001 (~52)	–	–	Two Power-law Functions
**Ficoll**	3.0 ± 0.1 (2.9–3.1)	1.5 +0.2/–0.1 (1.4–1.7)	0.010 ± 0.002 (~63)	–	–	Two Power-law Functions
**PEG**	2.7 ± 0.3 (2.4–3.0)	1.5 ± 0.1 (1.4–1.6)	0.009 ± 0.002 (~69)	–	–	Two Power-law Functions

In this hybrid model, polymer blobs are assumed as hard spheres, and their mutual exclusion due to the inter-polymer repulsive interactions yield the average center-to-center blob distance, extracted in the form of effective radius by fitting with the hybrid model. The profiles of the

effective concentration of PDADMAC in Ficoll (35 mg/mL) and PEG (12 mg/mL) environment, the low-q exponent is less reliable due to the limited fitting range. Therefore, we refrain from overinterpreting the absolute values of the low-q slopes. However, in all cases, the low-q scattering profiles in crowded environments indicate the presence of larger structures, despite the differences in the degrees of surface roughness.

Kratky Plots

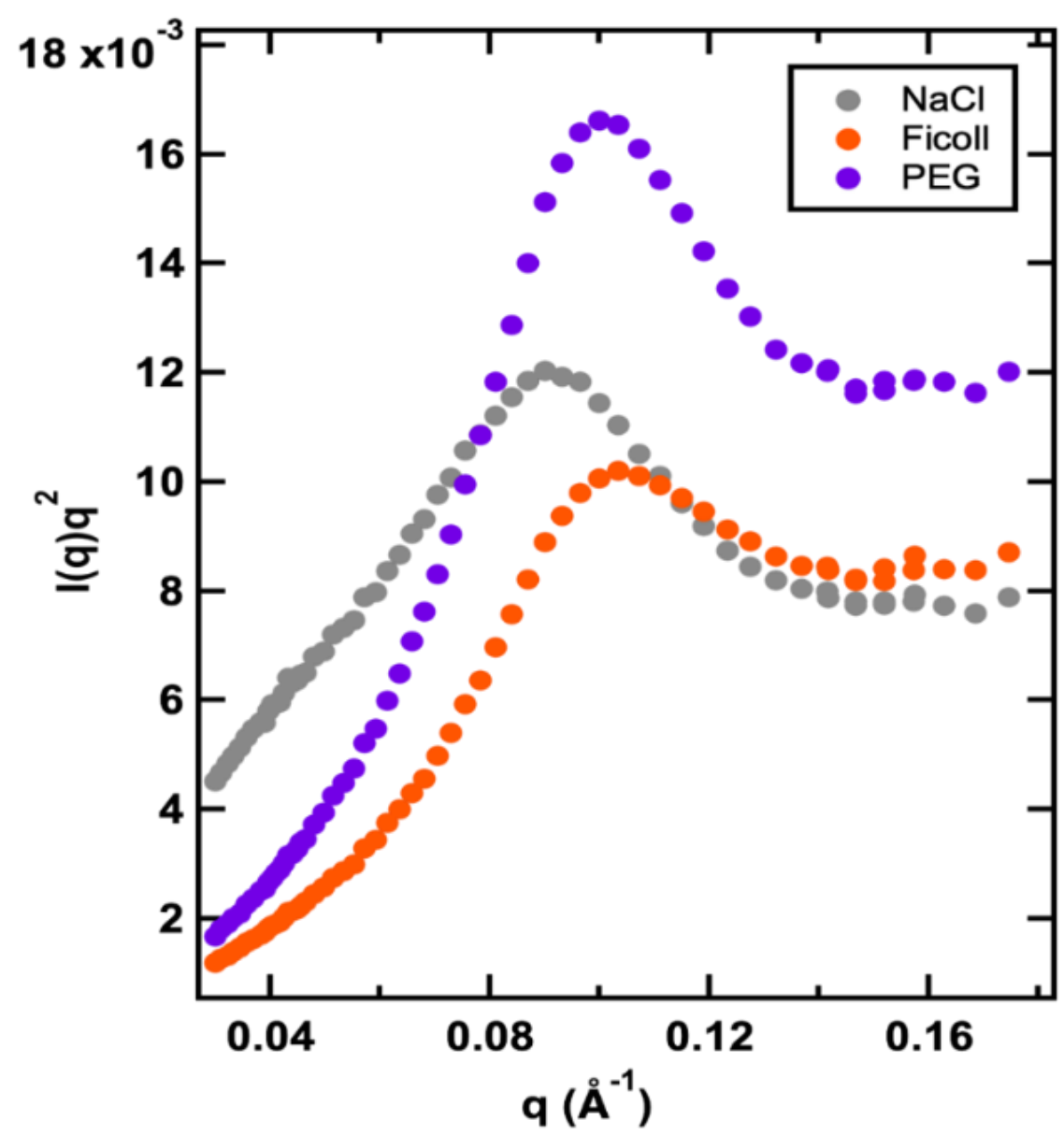

Figure S4. Kratky plots from SANS profiles measured from coacervate samples prepared in 100% D_2O in three solution conditions.

Turbidity and ζ-potential Measurements

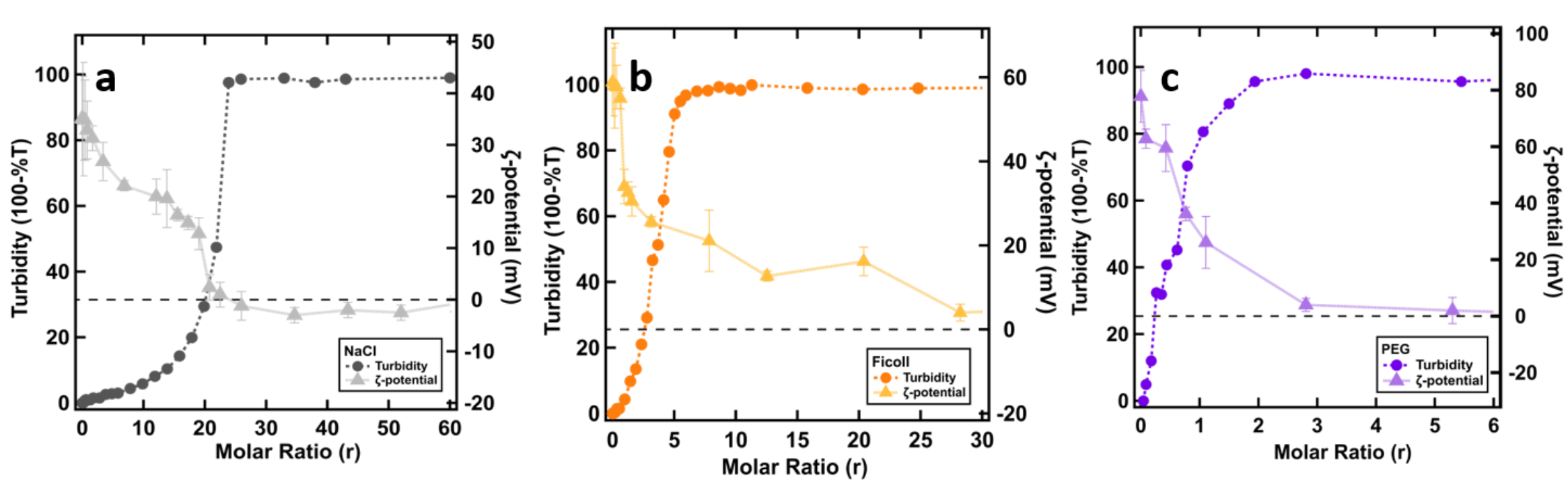

Figure S5. Turbidimetric titration and zeta potential measurements obtained from BSA (6 mg/mL) to PDADMAC (150 mg/mL) titration using concentrated protein and polymer solution. The titration was conducted in three different solution environments (a) NaCl (b) Ficoll-crowded environment, and (c) PEG-crowded environments.

FTIR and TGA of Complex Coacervate

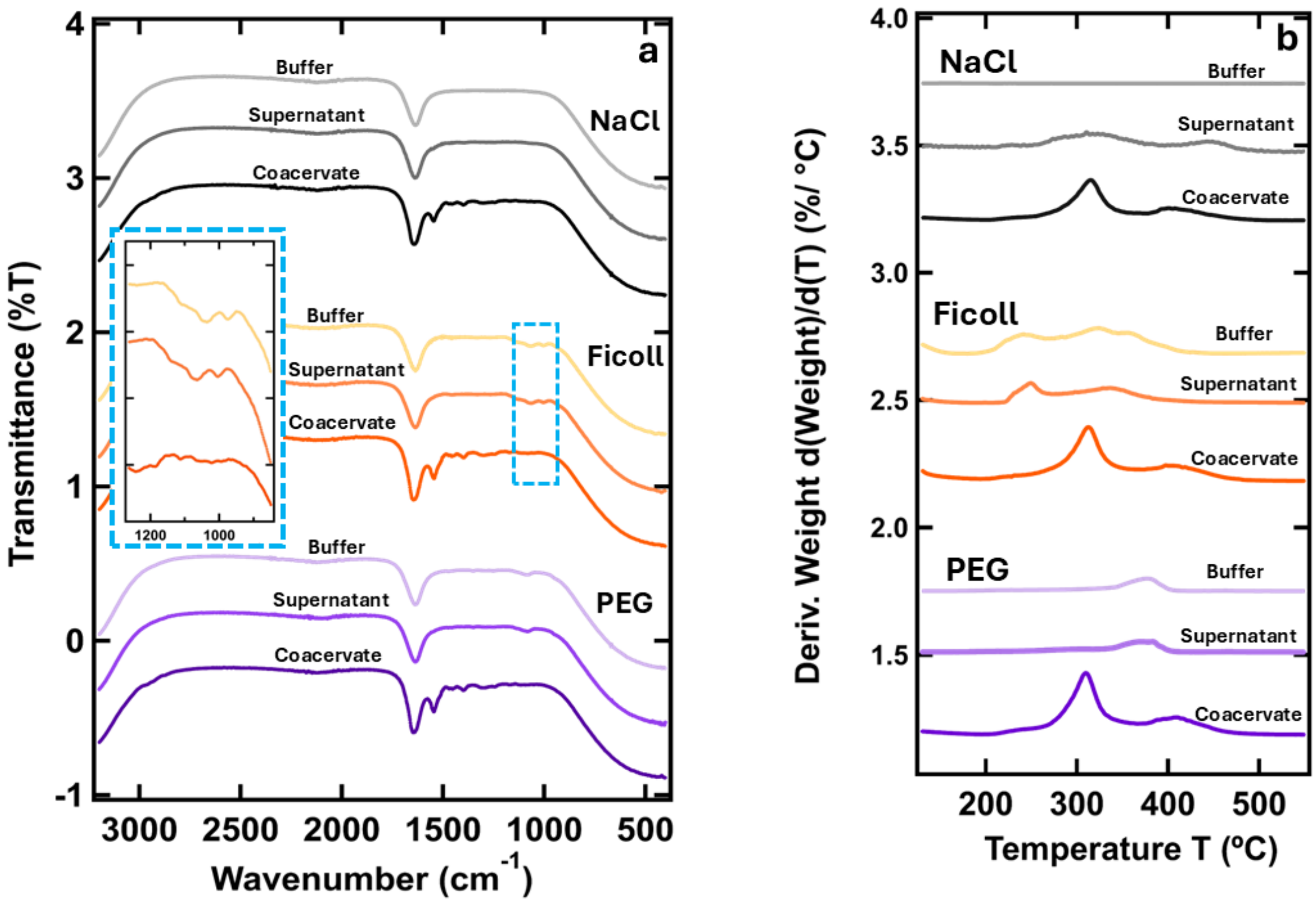

Figure S6. (a) FTIR spectra of buffer (light shade), supernatant (dark shade), and coacervate (darker shade) prepared in NaCl, Ficoll, and PEG solution conditions. Inset shows the magnified view of the 1300-900 cm^{-1} region (dashed blue box) for the Ficoll samples, highlighting differences in this region across different phases. (b) Differential thermogravimetric (DTG) plots of coacervate, supernatant, and buffers prepared in three solution conditions. 50 mM sNaCl as NaCl, PEG in 50 mM NaCl (PEG), and Ficoll in 50 mM NaCl (Ficoll) are referred to as buffers.

Rheological Measurement

The viscoelastic properties of the coacervates were characterized using a rheometer (HR20, TA Instruments, USA). All measurements were conducted with a stainless steel cone-and-plate geometry (40 mm diameter, 2° angle) at 25 °C, using a truncation gap of 53 µm considering the

viscous nature of the coacervate samples. To minimize solvent evaporation, a solvent trap was used during the experiments.

Strain sweep experiments were first conducted at a fixed angular frequency of 10 rad/s to determine the linear viscoelastic region (LVR) for each sample. Frequency sweep measurements were subsequently performed within the LVR at a low, constant strain amplitude of 4%, over an angular frequency range of 0.1–100 rad/s. All measurements were conducted in triplicate in order to ensure high reproducibility. The frequency dependence of the elastic modulus (G') is commonly used as an indicator of the viscoelastic nature of a gel[16, 17]. Thus, the frequency dependance of the storage (G') and loss (G'') modulus (Eq. S3 and S4) for coacervates were determined by power law model as follows[18, 19]:

$$G' = k'\omega^{p} \quad \text{Eq. S3}$$

$$G'' = k''\omega^{q} \quad \text{Eq. S4}$$

where k' and k''(Pa.s) are intercepts; p and q are the viscoelastic components of elastic and viscous moduli and ω is oscillatory frequency (Hz).To achieve a better quantitative understanding of the viscoelastic nature of the coacervates, the Winter Model[20] is applied to the complex viscosity data. The model predicts a power-law frequency dependence behavior given by-

$$\eta^{*} = S\omega^{n-1} \quad \text{Eq. S5}$$

Where S is the elastic strength of the material and n is the exponent indicative of the nature of cross-linking and varies between 1 for viscoelastic liquid at low frequency range and 0 for solid materials.

The $G^{"}$ versus frequency data were fitted using a power-law model, yielding slope values (n) of 0.8067, 0.9321, and 0.8876 for coacervates formed under NaCl, Ficoll, and PEG environments, respectively. These values indicate that coacervate is formed in NaCl is more liquid-like, while the most interconnected coacervate structure is observed under PEG and Ficoll-crowded conditions. Previous studies have shown that the slope values of G' and $G^{"}$ obtained in the frequency sweep measurements are influenced not only by the stoichiometric ratio but also by the concentration at which the interaction occurs in solution[21].

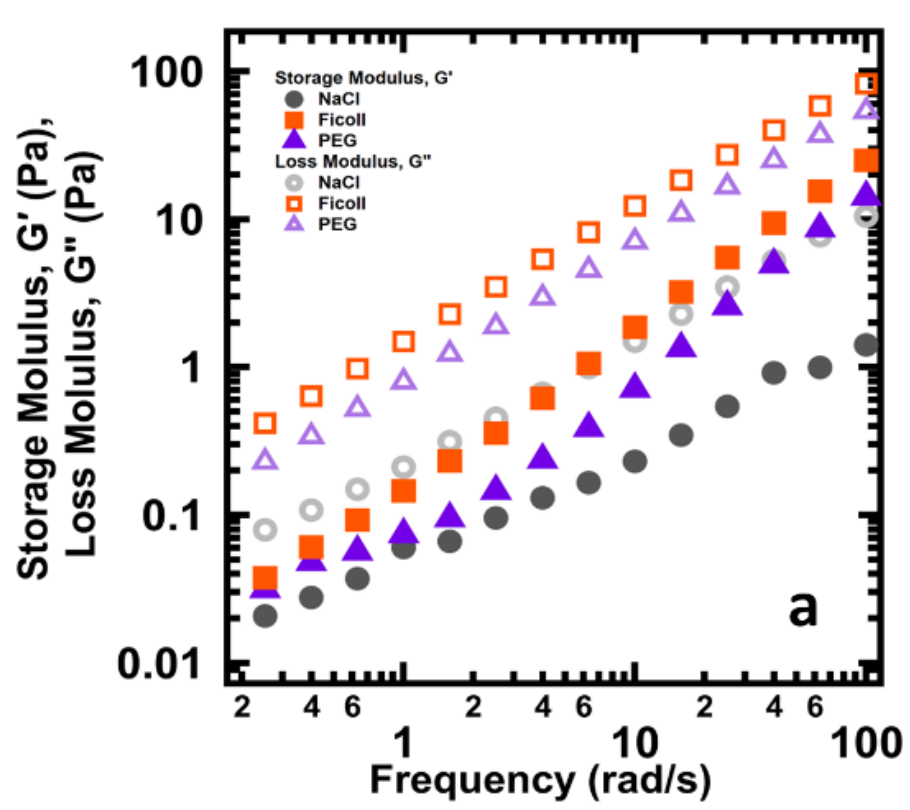

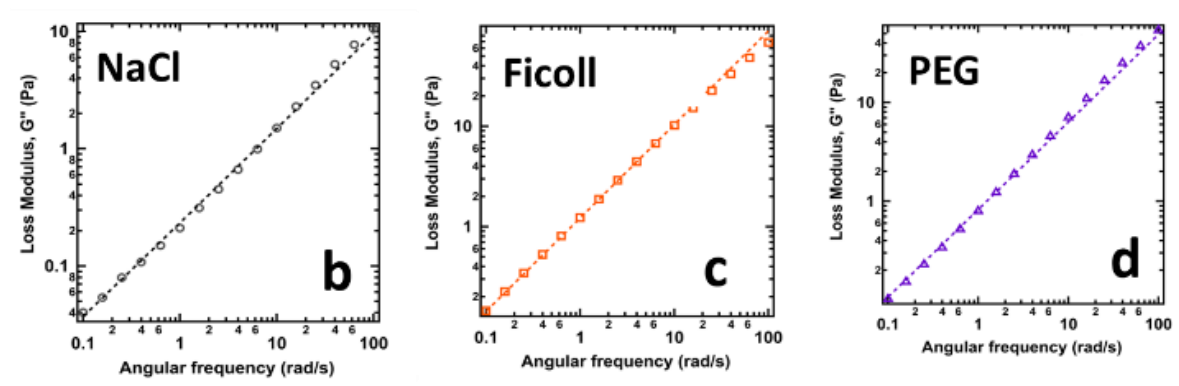

Table S2 *The parameters extracted from fitting the loss modulus vs frequency plots using Eq. S14.*

Coacervate	Exp of G”	R^2
NaCl	0.8067	0.992
Ficoll	0.9321	0.982
PEG	0.8876	0.991

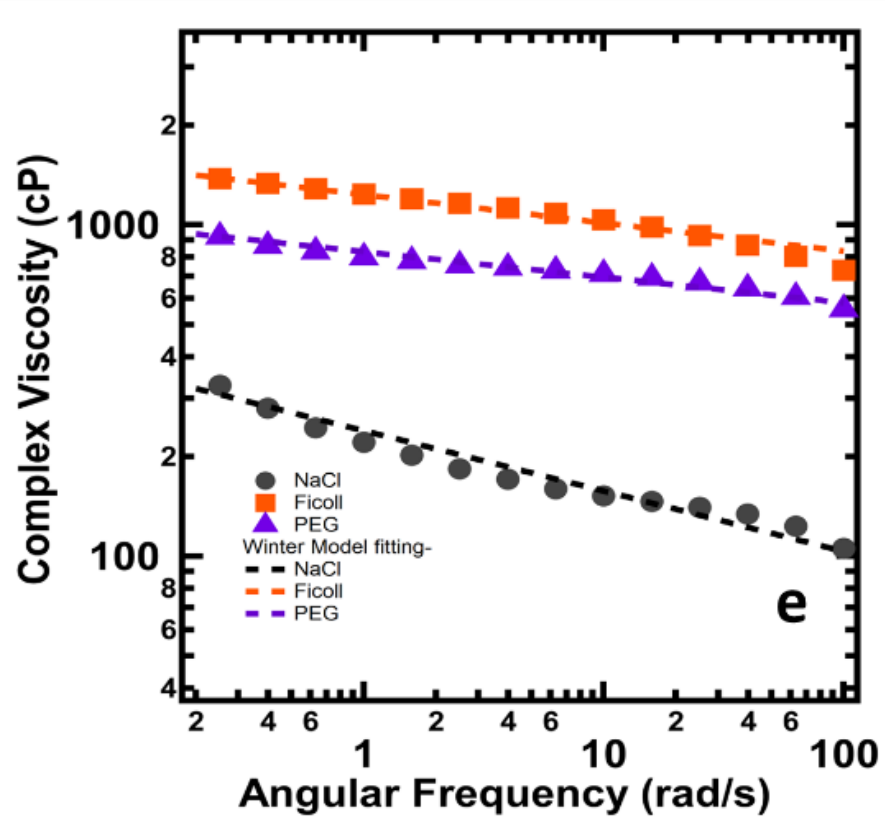

Table S3. *The parameters extracted from fitting complex viscosity vs frequency plots using Eq. S15.*

Coacervate	Slope of η*	*n*	*S*	R^2
NaCl	0.1821	0.8179	239.43	0.989
Ficoll	0.0782	0.9218	1233.7	0.991
PEG	0.0812	0.9188	830.49	0.990

Figure S7. (a) Storage and loss modulus Vs angular frequency for BSA-PDADMAC coacervates prepared under different solution conditions. Loss Modulus of complex coacervate in (b) NaCl, (c) Ficoll, and (d) PEG, fitted using power-law model. Table S2. shows the frequency dependencies of G” by a power law model for BSA/PDADAMC complex coacervates at various solution conditions. (e) Complex viscosity, η^* vs angular frequency plot of BSA-PDADMAC complex coacervates prepared at different solution environments. The broken lines define $\eta^* = S\omega^{n-1}$ (Winter model) fitting to the data points. The fitting parameters are shown in Table S3.

The variation of complex viscosity (η*) with angular frequency (ω) provides insight into the internal structure and dynamic response of coacervate under deformation (Figure S6). At low angular frequencies, the internal network structures of the system have sufficient time to reorganize and resist applied deformation while preserving structural integrity. In contrast, at high frequencies, limited relaxation time was provided to complete any structural rearrangement. Coacervates formed under Ficoll condition exhibit a nearly frequency-independent viscosity (slope of log η* vs. log ω = 0.0782), indicating a structurally robust internal network (Table S3). A similar trend is observed for coacervates formed in PEG (slope = 0.0812), suggesting comparable network stability under these conditions. In contrast, coacervate prepared in NaCl condition show a pronounced decrease in viscosity with increasing frequency (slope = 0.1821), consistent with shear-thinning behavior and comparatively more loosely organized internal network.

The complex viscosity (η*) versus frequency profiles were further analyzed using the Winter model, yielding the viscoelastic strength parameter (S) and power-law scaling exponent (n). Coacervates in Ficoll exhibited the highest S value (1233.7), consistent with the corresponding n values (Table S3), indicating enhanced network strength and structural robustness. A similar trend was observed in PEG, where η* decreased gradually with frequency, reflecting moderate viscoelastic stability. In contrast, coacervates in NaCl displayed a more pronounced decrease in viscosity, consistent with shear-thinning behavior and comparatively weaker structural integrity. Collectively, the combined S and n parameters indicate that coacervates formed in the presence of crowding agents exhibit higher resistance to deformation with increasing angular frequency compared to those formed under dilute condition. This relationship between complex viscosity and frequency is consistent with prior reports and further supports the shear-thinning nature of these coacervates [22, 23].

Microscopic Appearance of Coacervates under Different Experimental Conditions

Optical microscopy reveals that BSA-PDADMAC coacervates undergo distinct sedimentation-driven morphological evolution under dilute and crowded conditions, with the response governed by whether the sample is re-mixed after supernatant exchange (Figure S8).

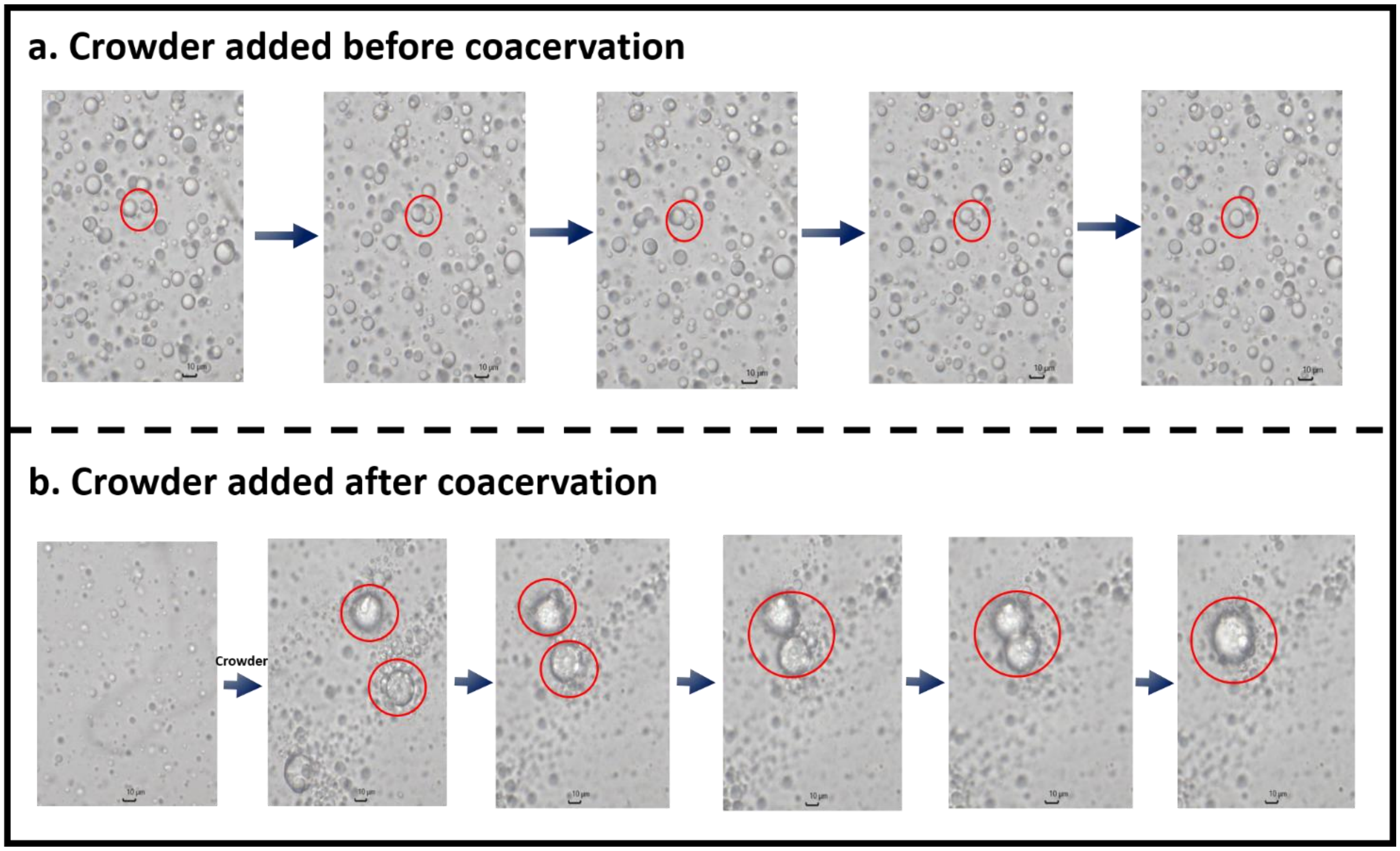

Figure S8. Formation of larger droplets upon addition of PEG (a) before, and (b) after BSA-PDADMAC coacervation. Similar results observed using Ficoll (not shown in this work). Scale bars: 10 μm.

In order to better understand the contribution of supernatants, we divided our experiments into two protocols. In the "With Re-mixing" condition, the supernatant obtained in dilute samples after 24 hours of gravitational sedimentation was exchanged with PEG solution, followed by re-mixing (Figure S9a) of the entire suspension to redistribute both the dense phase and dilute phases. In the "Without Re-mixing" condition, only the supernatant above the dense phase was carefully exchanged (Figure S9c) while leaving the bottom coacervate phase undisturbed.

In both approaches, samples were incubated for 24 hours at 4 °C before and after supernatant replacement. Same protocols for supernatant exchange experiment were followed for coacervate prepared in crowded conditions where the supernatant was replaced with NaCl solution (Figure S9b and S9d). In the re-mixed samples, replacing the supernatant with the solution of opposing types exhibits contrasting effects: droplets formed in dilute condition increase in size, whereas droplets found in crowded interior become smaller, indicating that the composition of the continuous phase strongly regulates the extent of droplet coarsening. Samples prepared "without re-mixing" exhibit the similar qualitative trend (Figure S9). Together, these results demonstrate that supernatant modulates both the extent and reversibility of sedimentation-driven droplet coarsening in BSA-PDADMAC coacervates. In addition to the droplet coalescence, supernatant exchange caused the droplet size distribution in crowded conditions to shift toward that observed under dilute, non-crowded conditions (Figure S9e and S9f), although this shift proceeded more gradually in the non-mixed system. This observation suggests that the two phases continue to exchange material even after macroscopic phase separation is complete, and that exchanging the supernatant pushes the system toward a new equilibrium reflecting the altered solution conditions.

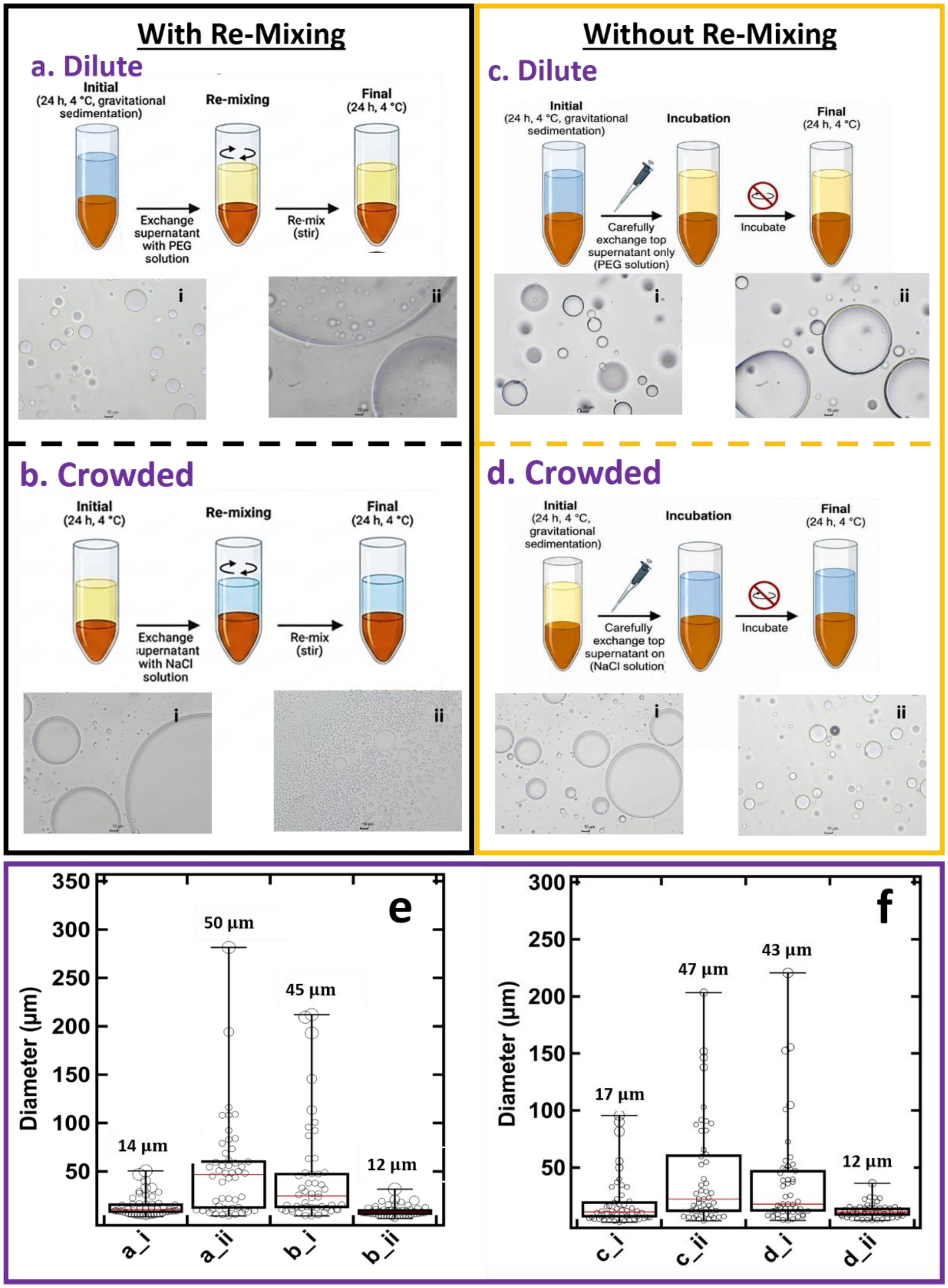

Figure S9. *Sedimentation-driven morphological evolution of BSA-PDADMAC coacervates as a function of supernatant-exchange protocol and solution condition. (a, b) Schematic of the "With Re-mixing" and (c, d) "Without Re-mixing" protocol. Corresponding bright-field images of BSA-PDADMAC complex coacervate prepared (a, b) "With" and (c, d) "Without" re-mixing are displayed within the black and yellow panel, respectively. (e, f) Quantification of droplet diameter for the "With Re-mixing" (e) and "Without Re-mixing" (f) conditions, comparing*

droplet size at the initial and final stage of supernatant exchange for dilute (a, c) and crowded (b, d) samples. Each open circle represents an individual droplet; boxes indicate the interquartile range, red lines denote the median, and whiskers span the full data range. The average droplet diameter for each condition is labeled adjacent to its corresponding box plot. Droplet diameters were measured from 50 droplets, collected from five micrographs acquired at different locations within the same sample. Scale bars, 10 μm.

REFERENCES

1. Nielsen, S.; Toft, K. N.; Snakenborg, D.; Jeppesen, M.; Jacobsen, J.; Vestergaard, B.; Kutter, J. P.; Arleth, L., BioXTAS RAW, a software program for high-throughput automated small-angle X-ray scattering data reduction and preliminary analysis. *J. Appl. Crystallogr.* **2009,** *42* (5), 959-964.
2. Kline, S. R., Reduction and analysis of SANS and USANS data using IGOR Pro. *J. Appl. Crystallogr.* **2006,** *39* (6), 895-900.
3. Sauter, A.; Zhang, F.; Szekely, N. K.; Pipich, V.; Sztucki, M.; Schreiber, F., Structural evolution of metastable protein aggregates in the presence of trivalent salt studied by (V) SANS and SAXS. *J. Phys. Chem. B* **2016,** *120* (24), 5564-5571.
4. Georgalis, Y.; Philipp, M.; Aleksandrova, R.; Krüger, J., Light scattering studies on Ficoll PM70 solutions reveal two distinct diffusive modes. *J. Colloid Interface Sci.* **2012,** *386* (1), 141-147.
5. Kayitmazer, A. B.; Bohidar, H. B.; Mattison, K. W.; Bose, A.; Sarkar, J.; Hashidzume, A.; Russo, P. S.; Jaeger, W.; Dubin, P. L., Mesophase separation and probe dynamics in protein–polyelectrolyte coacervates. *Soft Matter* **2007,** *3* (8), 1064-1076.
6. Ranganathan, V. T.; Bazmi, S.; Wallin, S.; Liu, Y.; Yethiraj, A., Is ficoll a colloid or polymer? A multitechnique study of a prototypical excluded-volume macromolecular crowder. *Macromolecules* **2022,** *55* (20), 9103-9112.
7. Sajid, A.; Castronovo, M.; Goycoolea, F. M., On the Fractionation and Physicochemical Characterisation of Self-Assembled Chitosan–DNA Polyelectrolyte Complexes. *Polymers* **2023,** *15* (9), 2115.
8. Poudyal, M.; Patel, K.; Sawner, A. S.; Gadhe, L.; Kadu, P.; Datta, D.; Mukherjee, S.; Ray, S.; Navalkar, A.; Maiti, S., Liquid condensate is a common state of proteins and polypeptides at the regime of high intermolecular interactions. *BioRxiv* **2022**, 2021.12. 31.474648.
9. Ou, X.; Tang, Z.; Ye, Y.; Chen, X.; Huang, Y., Macromolecular Crowding Effect on Chitosan-Hyaluronic Acid Complexation and the Activity of Encapsulated Catalase. *Biomacromolecules* **2024**.
10. Patel, C. K.; Singh, S.; Saini, B.; Mukherjee, T. K., Macromolecular crowding-induced unusual liquid–liquid phase separation of human serum albumin via soft protein–protein interactions. *J. Phys. Chem. Lett.* **2022,** *13* (16), 3636-3644.
11. Niezabitowska, E.; Town, A. R.; Sabagh, B.; Moctezuma, M. D. M.; Kearns, V. R.; Spain, S. G.; Rannard, S. P.; McDonald, T. O., Insights into the internal structures of nanogels using a versatile asymmetric-flow field-flow fractionation method. *Nanoscale Adv.* **2020,** *2* (10), 4713-4721.
12. Jia, D.; Muthukumar, M., Dipole-driven interlude of mesomorphism in polyelectrolyte solutions. *Proceedings of the National Academy of Sciences* **2022,** *119* (40), e2204163119.

13. Pamvouxoglou, A.; Bogri, P.; Nägele, G.; Ohno, K.; Petekidis, G., Structure and dynamics in suspensions of soft core-shell colloids in the fluid regime. *The journal of chemical physics* **2019,** *151* (2).
14. Li, J.; Jiang, X.; Singh, A.; Heinonen, O. G.; Hernández-Ortiz, J. P.; de Pablo, J. J., Structure and dynamics of hydrodynamically interacting finite-size Brownian particles in a spherical cavity: Spheres and cylinders. *Proc. Natl. Acad. Sci.* **2020,** *152* (20).
15. Riest, J.; Eckert, T.; Richtering, W.; Nägele, G., Dynamics of suspensions of hydrodynamically structured particles: analytic theory and applications to experiments. *Soft Matter* **2015,** *11* (14), 2821-2843.
16. Ikeda, S.; Foegeding, E. A.; Hagiwara, T., Rheological study on the fractal nature of the protein gel structure. *Langmuir* **1999,** *15* (25), 8584-8589.
17. Ikeda, S.; Foegeding, E., Dynamic viscoelastic properties of thermally induced whey protein isolate gels with added lecithin. *Food Hydrocolloids* **1999,** *13* (3), 245-254.
18. Ramkumar, D.; Vaidya, U.; Bhattacharya, M.; Hakkarainen, M.; Albertsson, A.-C.; Karlsson, S., Properties of injection moulded starch/synthetic polymer blends—I. Effect of processing parameters on physical properties. *Eur. Polym. J.* **1996,** *32* (8), 999-1010.
19. Khondkar, D.; Tester, R. F.; Hudson, N.; Karkalas, J.; Morrow, J., Rheological behaviour of uncross-linked and cross-linked gelatinised waxy maize starch with pectin gels. *Food Hydrocolloids* **2007,** *21* (8), 1296-1301.
20. Winter, H. H., Can the gel point of a cross-linking polymer be detected by the G′–G ″crossover? *Polym. Eng. Sci.* **1987,** *27* (22), 1698-1702.
21. Scanlan, J. C.; Winter, H. H., Composition dependence of the viscoelasticity of end-linked poly (dimethylsiloxane) at the gel point. *Macromolecules* **1991,** *24* (1), 47-54.
22. Bastos, D. S.; Barreto, B. N.; Souza, H. K.; Bastos, M.; Rocha-Leão, M. H. M.; Andrade, C. T.; Gonçalves, M. P., Characterization of a chitosan sample extracted from Brazilian shrimps and its application to obtain insoluble complexes with a commercial whey protein isolate. *Food Hydrocolloids* **2010,** *24* (8), 709-718.
23. Wang, X.; Lee, J.; Wang, Y.-W.; Huang, Q., Composition and rheological properties of β-lactoglobulin/pectin coacervates: Effects of salt concentration and initial protein/polysaccharide ratio. *Biomacromolecules* **2007,** *8* (3), 992-997.
